# A novel Gravity-FREAK feature extraction and Gravity-KLT tracking registration algorithm based on iPhone MEMS mobile sensor in mobile environment

**DOI:** 10.1371/journal.pone.0186176

**Published:** 2017-10-31

**Authors:** Zhiling Hong, Fan Lin, Bin Xiao

**Affiliations:** Software School, Xiamen University, Xiamen, Fujian, China; Tianjin University, CHINA

## Abstract

Based on the traditional Fast Retina Keypoint (FREAK) feature description algorithm, this paper proposed a Gravity-FREAK feature description algorithm based on Micro-electromechanical Systems (MEMS) sensor to overcome the limited computing performance and memory resources of mobile devices and further improve the reality interaction experience of clients through digital information added to the real world by augmented reality technology. The algorithm takes the gravity projection vector corresponding to the feature point as its feature orientation, which saved the time of calculating the neighborhood gray gradient of each feature point, reduced the cost of calculation and improved the accuracy of feature extraction. In the case of registration method of matching and tracking natural features, the adaptive and generic corner detection based on the Gravity-FREAK matching purification algorithm was used to eliminate abnormal matches, and Gravity Kaneda-Lucas Tracking (KLT) algorithm based on MEMS sensor can be used for the tracking registration of the targets and robustness improvement of tracking registration algorithm under mobile environment.

## Introduction

**Fig 1:**
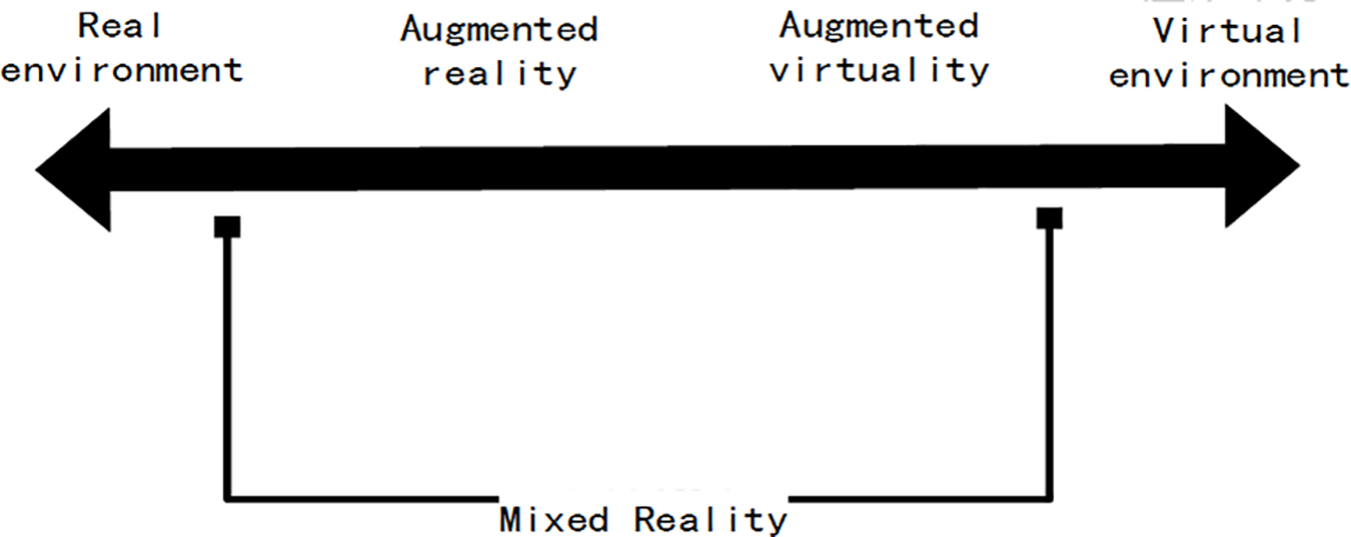
Mixed reality.

With the rapid development of image processing and artificial intelligence, the conception can be realized through the combing use of different technologies and the augmented reality technology which focuses on virtual-real fusion emerged [1,36,39]. Different from the virtual reality technologies that focus on introducing users to virtual 3D scenes, the augmented reality technology emphasizes how to accurately integrate the virtual information generalized by computer into the real-world environment so that to realize the simultaneous presentation of virtual information and the real environment for the supplementation and enhancement of the real environment. The relationship between the two parts is show as Fig 1:

Generally, the augmented reality system is consisted of three parts: virtual-real fusion, real-time interaction and 3D registration [2]. Among the three parts, 3D registration, the accurate matching between virtual and real environments, is the key restraining factor of wider application of augmented reality technology. Most of the traditional 3D registration methods were designed and proposed on the basis of PC [3,38]. They cannot be applied to mobile augmented reality systems directly as most of the mainstream mobile devices are not equipped with floating point processor (FPP), and the CPU speed and memory capacity are not able to support the devices efficiently to conduct feature extraction and position calculation of the target. Hence, it becomes an urgent matter to search a mobile 3D registration algorithm with better performance and lower resource occupation to popularize mobile augmented reality.

## Related work

As the product of the constant development of virtual reality technology, the appearance of augmented reality can be traced back to the HMD (Head Mounted Display) invented by an American in 1965 [4]. Through the device, the user can visualize the superposition of real environment and 3D image. Until the early 1990s, the concept of augmented reality was first proposed by Caudell and Mizell [5], scientists from Boeing Co. After that, the size of portable device became smaller and smaller, while the computing performance became stronger and stronger, which makes it possible to conduct image rendering and superposition on mobile devices. In 1997, Feiner et al. [6] designed the first prototype of mobile augmented reality system. The system can add 3D travel guide information onto the real built environment. By the end of 1990s, augmented reality became an independent and significant research field which attracted more and more researchers. Many AR related international conferences also emerged, such as IWSAR (International Workshop and Symposium on Augmented Reality), ISMR (International Symposium on Mixed Reality), DARE (Designing Augmented Reality Environments workshop), etc. Among all the research directions, the research on AR tracking registration technology is always the hotspot, which is also the key step in the application of AR. According to the registration method, AR system can be divided into sensor oriented system and machine vision oriented system.

### Sensor oriented tracking registration

Sensor oriented tracking method has long ago been applied to AR registration field by researchers, including mechanical tracking registration, electromagnetic tracking registration, ultrasonic tracking registration, GPS tracking registration and inertial tracking registration, etc. The method replies on the related sensor function of the hardware device. With the accurate real-time data provided by the sensor, the method can obtain the position and direction information of the tracking target. The outdoor AR system designed by Feiner et al. [6] used sensors as GPS and angle instrument for tracking registration. However, the method has high requirements of hardware and environment. Many sensor oriented tracking registration methods are still in the experimental stage, which cannot be promoted to ordinary users.

### Computer vision oriented tracking registration

Compared with sensor oriented tracking registration, the computer vision oriented method has a wider practicability. Based on different identification method, the computer vision oriented method can be divided into artificial identification oriented registration method and natural feature oriented registration method.

The artificial identification oriented registration method needs to install landmark with obvious identification feature in natural environment, which can be identified in the video image through matching algorithm. With regular geometrical features and known position in the natural environment coordinate system, the landmark can be captured easily and accurately by computer. Then the camera can calibrate the algorithm and obtain the accurate parameters of the projection matrix. In this way, the virtual 3D image can be superimposed on the position of the real environment landmark [7–11]. Without any requirement of rebuilding natural environment with small computational amount, this method is proper for the mobile devices with limited computing and main memory resources. However, due to the unsatisfactory performance of matching algorithm in light and shad robustness, it is hard to be applied in real application environment on a large scale.

**Fig 2:**
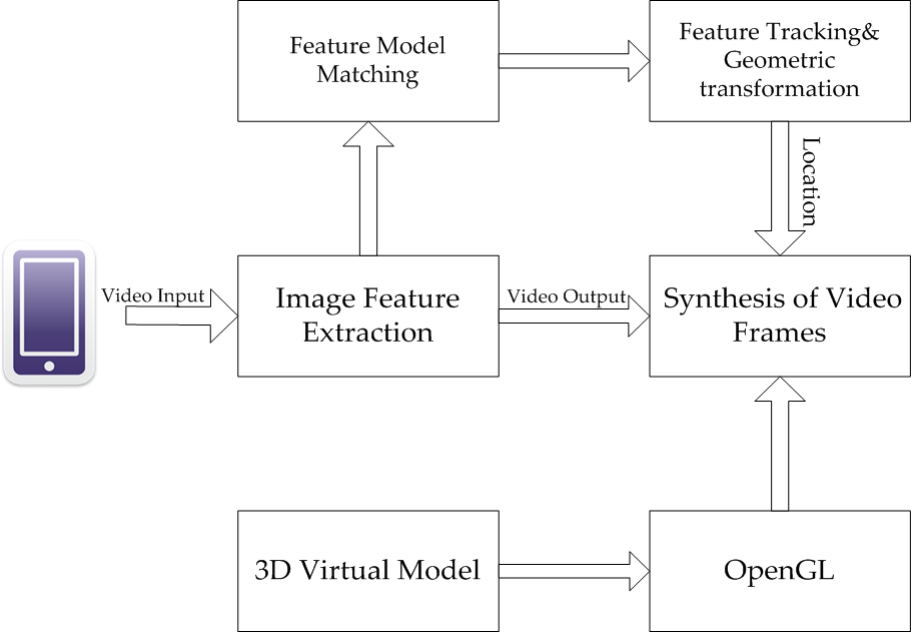
The framework of mobile augmented reality based on natural feature extraction.

The natural feature oriented registration method uses natural feature to conduct 3D registration, mainly using vision positioning technology and image extraction algorithm to realize an accurate positioning and tracking of the target for 3D registration, as shown in Fig 2. Traditional vision feature tracking methods included: EKF (Extended Kalman Filter) [12] method and systems based on particle filter [13] and unscented Kalman filter [14]. On account of SLAM, Klein.G, et al. proposed the augmented reality system based on parallel tracking and mapping (PTAM) [15]. The method was applied in mobile devices by Klein.G, et al. [16] in 2009.

There are many methods of feature point extraction and description. The mostly common used methods include FAST, SIFT, and SURF, etc. In 2010, Wagner improved SIFT and Ferns [17,18] by replacing the DOG feature detecting algorithm in SIFT with FAST, reduced the number of feature vector dimensions and realized the 6-DoF real-time mobile augmented reality system on smartphones.

## Gravity-FREAK feature extraction and description

On account of the performance problems on mobile devices of feature description algorithms as SIFT, SURF and ORB, this paper used AGAST algorithm to extract feature points and proposed an improved FREAK feature description algorithm combining with the inertial sensor commonly equipped on mobile devices [19–22,35,37].

### AGAST

As a feature point extraction algorithm proposed by Mair et al. [19,20], AGAST added scale information onto the feature point on the basis of FAST algorithm, which makes the algorithm more accurate in the positioning performance. In order to obtain high quality feature points with scale information, AGAST algorithm not only conducted the extremum search in its image space, but also used FAST algorithm to detect the extremum at each scale space.

Before the extremum detecting, n octaves *c*_*i*_ and n intra-octaves *d*_*i*_ should be constructed to compose their solution pyramid needed for the research. Normally *i* = {0,1,2,⋯,*n*−1}, and n = 4. Octaves *c*_*j*_ can be obtained through semi-sampling of *c*_*j*−1_, and *c*_0_ is the original image. Interlayer *d*_*i*_ is located in the middle of *c*_*i*_ and *c*_*i*+1_. The first interlayer was generated through the down sampling with the parameter of 1.5 of original image, and the rest interlayers were obtained through the semi-sampling of the previous interlayer as shown in Formula 1:

$$\left\{ \begin{array}{l} t(c_{i})=2^{i}\\ t(d_{i})=2^{i}\cdot 1.5 \end{array}\right.$$ (1)

Where *t* refers to the image dimension.

**Fig 3:**
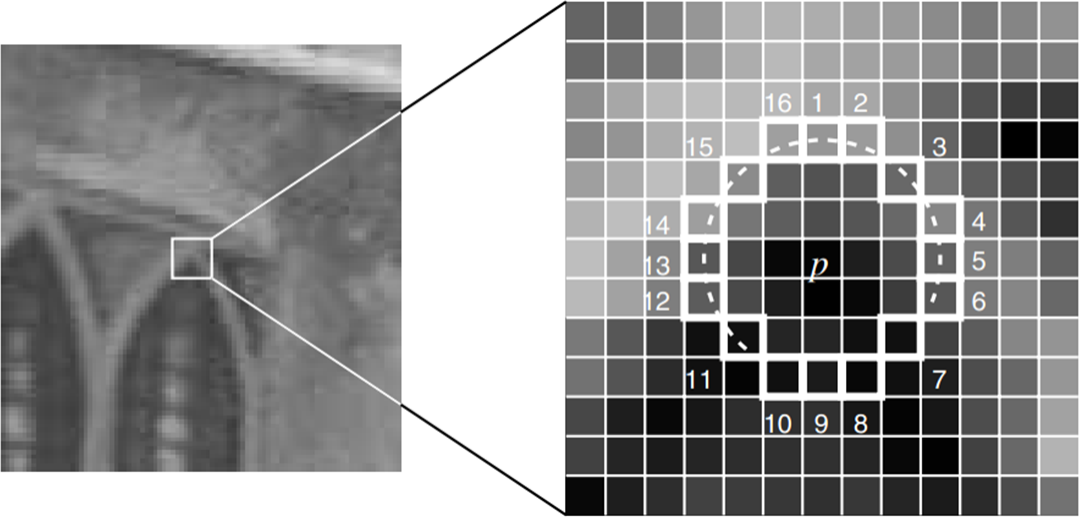
FAST 9–16 working principle diagram.

In the extremum test, FAST 9–16 template is often used first to extract the potential interest points of each octave and the middle octave. As shown in Fig 3, the white dash line is a circle taking the key point to be tested p as the center and 3 pixels are the radius with totally 16 pixels. According to the requirement of the test standard, only if the brightness of at least 9 pixels of all the 16 pixels is higher or lower than that of the key point and exceeds the threshold value t, the key point can be determined as the potential feature point as shown in Formula 2:

$$\left\{ \begin{array}{l} V=\max(\sum_{x\in S_{bright}}\left|I_{p\to x}-I_{p}\right|-t,\sum_{x\in S_{dark}}\left|I_{p}-I_{p\to x}\right|-t)\\ S_{bright}=\left\{x|I_{p\to x}\geq I_{p}+t\right\}\\ S_{dark}=\left\{x|I_{p\to x}\leq I_{p}-t\right\} \end{array}\right.$$ (2)

Where V is the FAST 9–16 score of the key point.

Then the obtained potential interest point needs to be conducted with non-maximum suppression. The real feature point needs to satisfy the following two conditions: (1) In the area of the octave with the point as the center, its FAST score should be the highest among the 9 points; (2) In the corresponding upper and lower areas, its FAST score should be the highest. The extremum test on the octave *c*_0_ is an exception: FAST 5–8 template is used on *c*_0_ for calculation as the FAST score on the virtual middle octave d-1 needs to be captured.

### Traditional FREAK feature description

FREAK algorithm is a binary feature description algorithm proposed by the study of Alahi.A, et al. in 2012. The core idea of FREAK algorithm is to use the sampling template of imitated retinal structure to conduct the descriptor structure of feature point.

Similar to the descriptor of image feature as ORB, FREAK constructs the binary descriptor F by calculating the difference of Gaussian convolution (DoG) at a sampling point pair as shown in Formula 3:

$$F=\sum_{0\leq a\leq N}2^{a}T(P_{a})$$ (3)

where *P*_*a*_ refers to a sampling point pair, N stands for the bit length of descriptor, and *T*(*P*_*a*_) satisfies Formula 4:

$$T(P_{a})=\left\{ \begin{array}{ll} 1 & if(I(P_{a}^{r_{1}})-I(P_{a}^{r_{2}})>0)\\ 0 & otherwise \end{array}\right.$$ (4)

where $I(P_{a}^{r_{1}})$ refers to the brightness of the first sampling point in *P*_*a*_.

**Fig 4:**
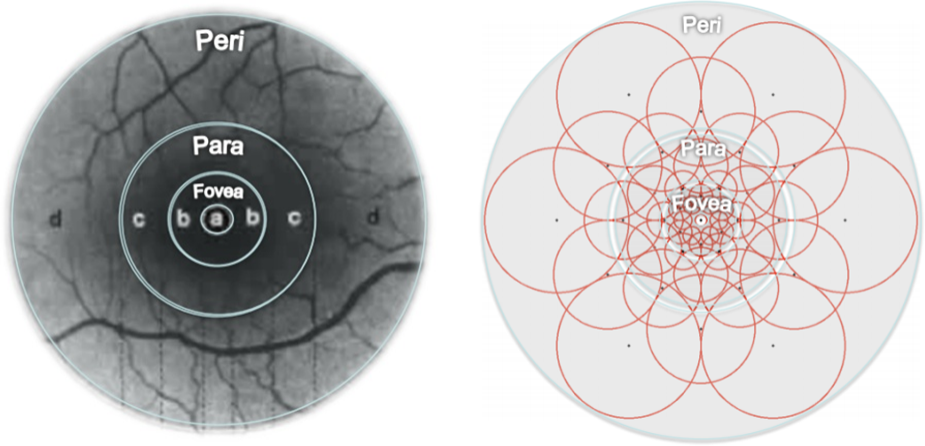
Diagram of human retina structure (left) and FREAK sampling template (right).

Generally, if 43 sampling areas were selected in the neighboring area of the feature point as shown in Fig 4 (right), a single feature point would generate almost one thousand sampling point pairs. However, the factor is that only some of the point pairs can effectively describe the image information. Hence, FREAK designed an algorithm based on ORB descriptor to select the best sampling point pair from the training set.

**Fig 5:**
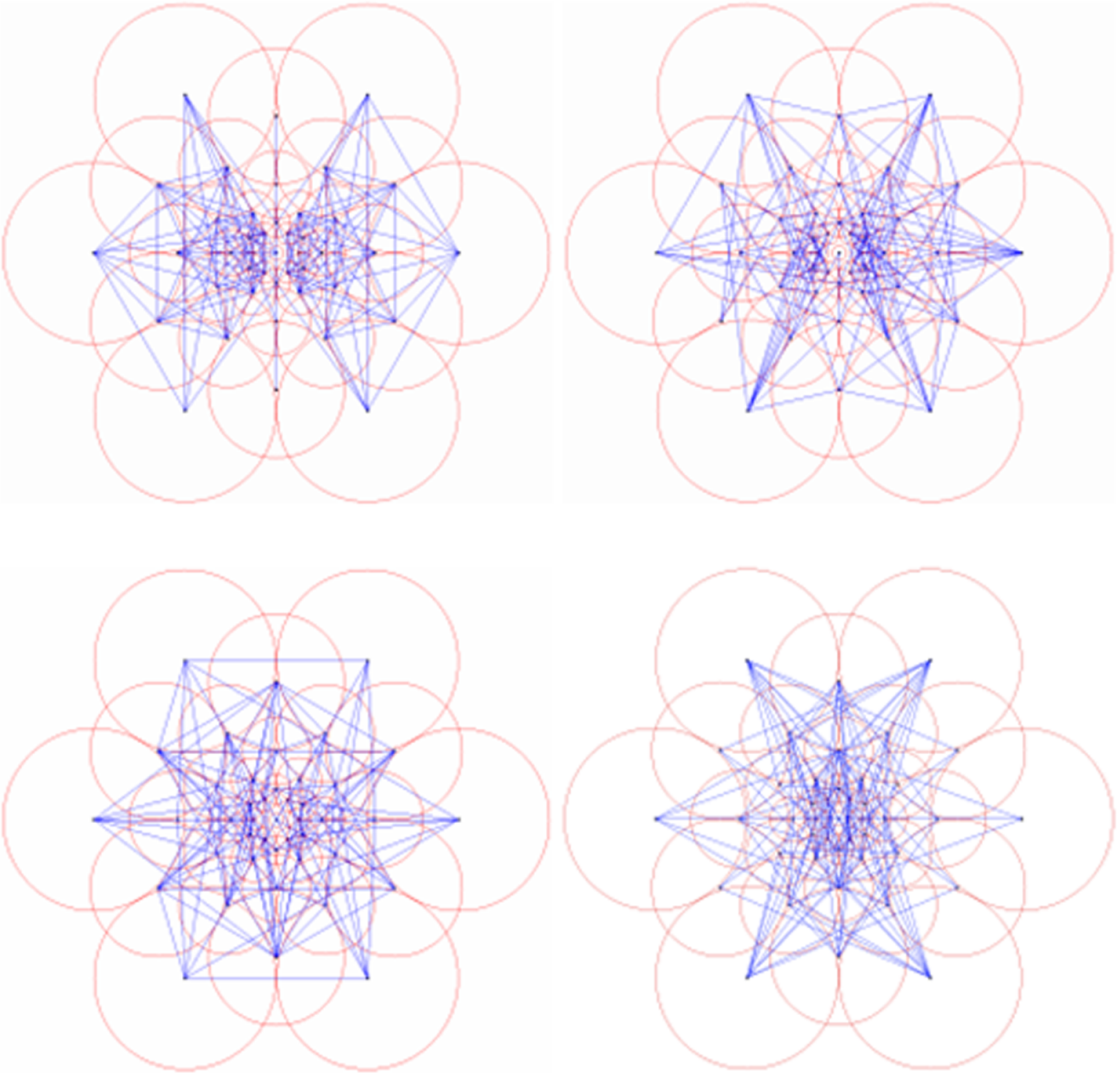
Sampling point pair from coarse to fine.

The 512 sampling point pairs selected by above algorithm already embraced the idea of arrangement from rough to fine of Gaussian difference. As shown in Fig 5, if the 512 sampling point pairs were divided into 4 groups to 128 pairs a group, the 4 groups can happen to be corresponding to the 4 areas of human retina. The first group of sampling point pairs is mostly located at lateral side corresponding to the perifoveal area of retina, while the last group of sampling point pairs is mostly located at center area corresponding to the foveal area of retina. Specifically, the first 16 bytes of the FREAK descriptor can be used for the coarse-grained comparison. If the matching distance is less than a threshold value, keep using the rest bytes for more sophisticated comparison. The cascade method greatly accelerated the matching speed of descriptor as 90% of the candidates have been abandoned in the previous 16 bytes screening.

**Fig 6:**
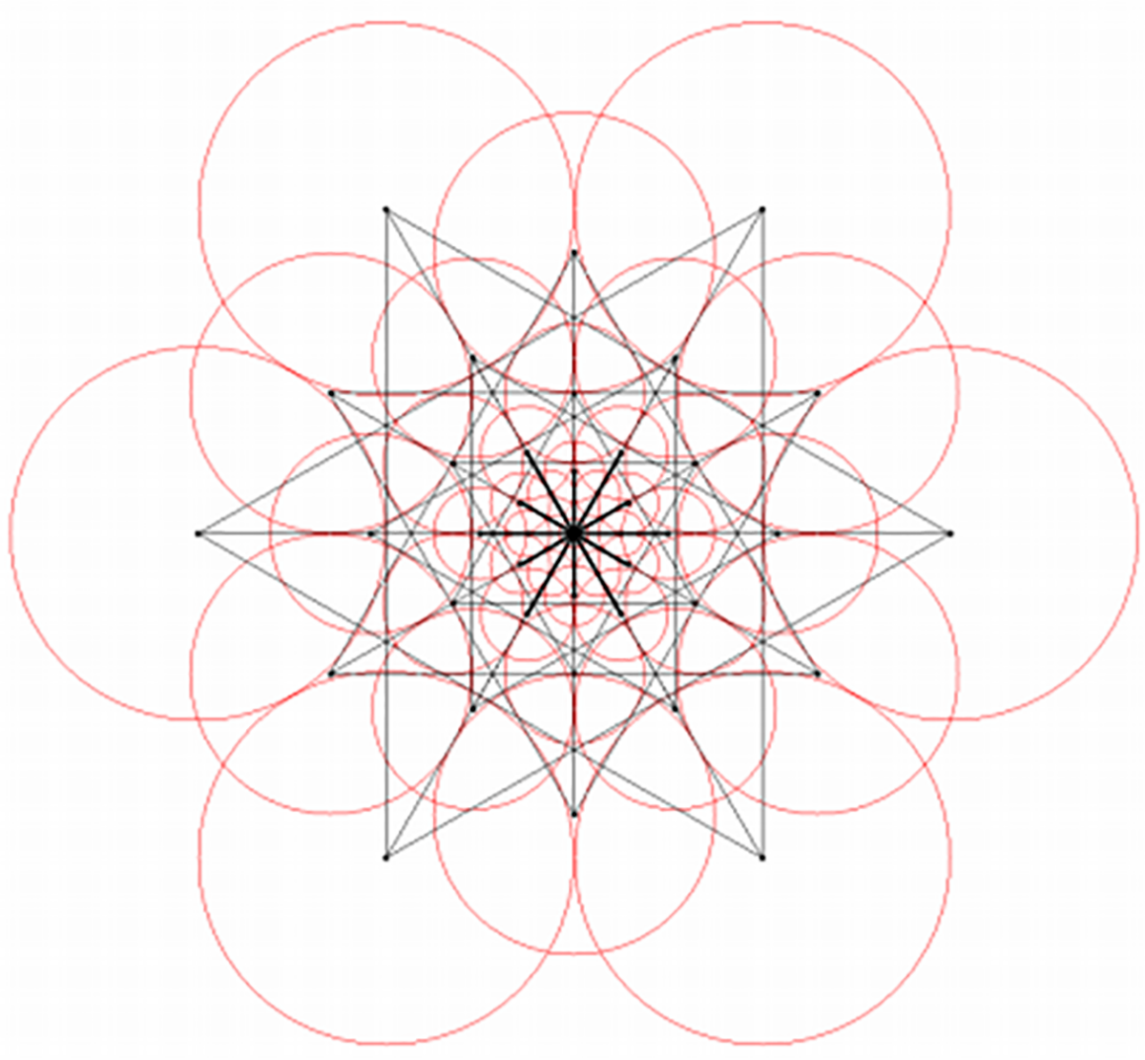
Sampling point pair of calculating feature direction.

In addition, to estimate the direction of feature point, FREAK used similar descriptor method as BRISK to calculate the local gradient. The difference is the FREAK uses centrosymmetric sampling area as shown in Fig 6:

G is assumed as the set consisted by all the sampling point pairs calculating the local gradient. The calculating formula of feature point direction is shown as Formula 5:

$$O=\frac{1}{M}\sum_{P_{o}\in G}(I(P_{o}^{r_{1}})-I(P_{o}^{r_{2}}))\frac{P_{o}^{r_{1}}-P_{o}^{r_{2}}}{\left\|P_{o}^{r_{1}}-P_{o}^{r_{2}}\right\|}$$ (5)

where M refers to the element number of set G; $P_{o}^{r_{i}}$ represents the two-dimensional vector of sampling center.

Comparing with the requirement of more than 100 sampling point pairs of BRISK, the calculation of FREAK feature direction only needs 45 point pairs. Besides, FREAK retina sampling template has a larger sampling area in perifoveal area, which can better calculate the deviation from redundant direction and has lower memory occupation.

### FREAK description based on gravity

At present, mainstream mobile devices are all equipped with gravity sensor. Combined with computer vision and MEMS sensor, the direction gesture information obtained by AR system is more accurate and stable. As early as 2000, S. You et al. [23] already used the mixed method of inertial sensor and computer vision to conduct target tracking. In order to improve the feature matching of panoramic picture, Kurz D. and Himane S. B. (2011) [33]used gyroscope to measure the direction and posture variation when the camera shooting two adjacent frames. The second image will be affine transformed to align with the first image. Then the SIFT feature point of the two images will be extracted for the matching.

#### Gravity model of mobile device

**Fig 7:**
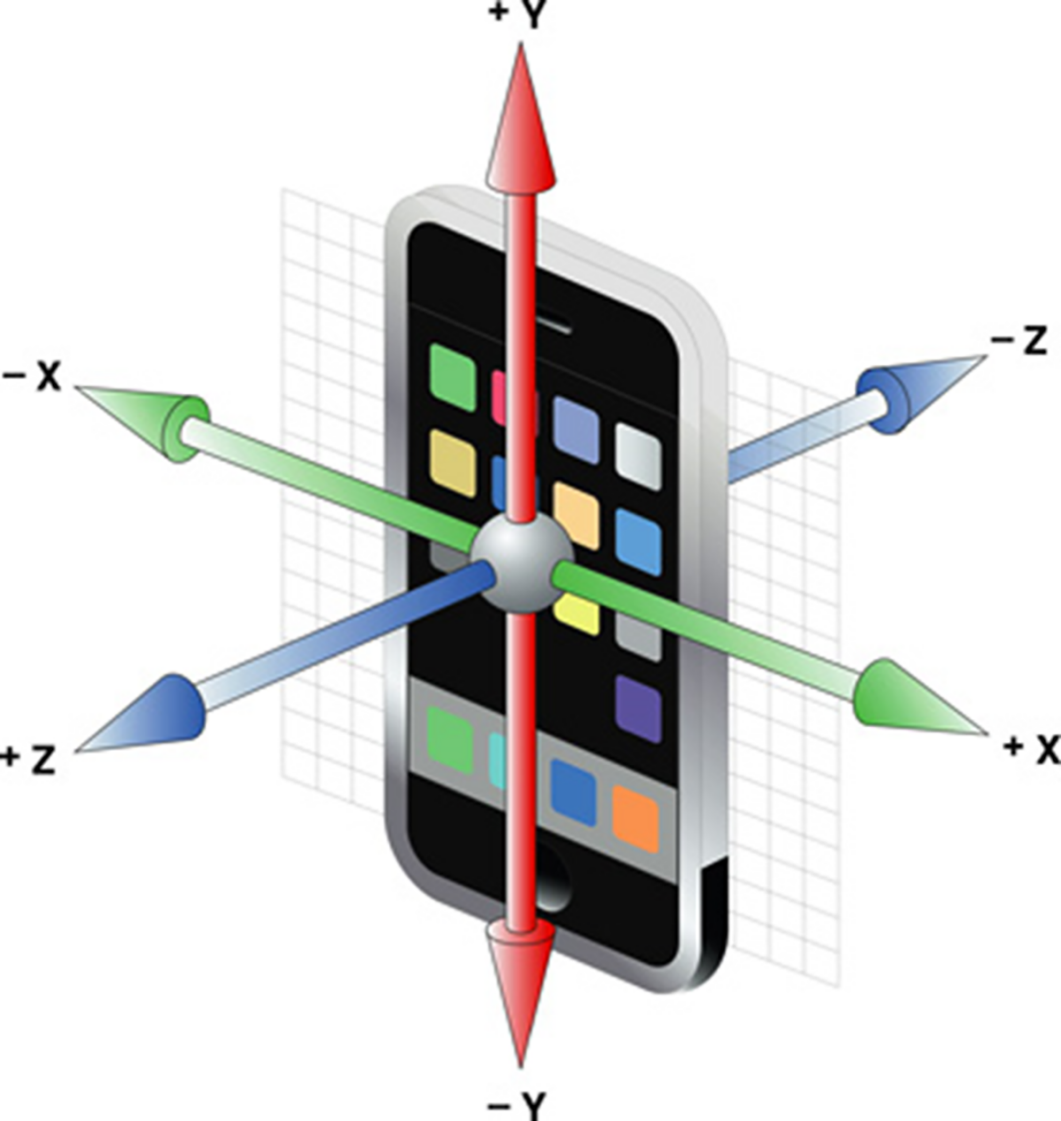
Diagram of the three acceleration component direction of smartphone.

In order to obtain the current gravity direction of a point in the image, gravity sensor will be used to test and record the position and posture of the camera when shooting. Take Apple iOS system for example, the Core Motion Framework frame already encapsulated the related operations of the acceleration sensor into the UI Acceleration category and eliminated the impacts of user’s shaking on current posture of the device by combining with the data from the gyroscope. The instance object of the category can be created to directly read x, y, z attributes to estimate the current position and posture. As shown in Fig 7, x, y, z represents the instantaneous acceleration component of the mobile device in the three directions respectively.

Acceleration component is a double type data type, with the value range from -1 to +1 and gravity unit of g. For example, if the value is 1.0, the acceleration of the device along the direction equals to a gravity unit. When an iPhone was placed still on a horizontal surface with the screen upward, the current output acceleration value approximates as.

As the gravity effect at each point on the imaging plane is uneven, the 2D projection of the 3D space gravity on the image plane needs to be calculated to accurately mark the gravity direction of the current image feature point [33]. Given the normalized vector of 3D space gravity *g* = [*g*_*x*_,*g*_*y*_,*g*_*z*_]^*T*^, g satisfying the condition of ‖*g*‖ = 1, and given the camera internal parameter K (matrix), then 2D projection vector *d* = [*d*_*u*_,*d*_*v*_,0]^*T*^ of the actual environmental gravity at the pixel point *p* = [*u*,*v*,1]^*T*^ can be calculated through Formula 6[33]:

d=p'−p (6)

Where *p*' = [*u*',*v*',1]^*T*^, can be obtained by Formula 7[33]:

$${\left[(1+g_{x})u',(1+g_{y})v',(1+g_{z})\right]}^{T}=p+Kg$$ (7)

As the length of vector d and g can be any value, then [33]:

$$d\propto {\left[g_{z}(p_{u}-u)+f_{u}g_{x},g_{z}(p_{v}-v)+f_{v}g_{y},0\right]}^{T}$$ (8)

Where [*p*_*u*_,*p*_*v*_]^*T*^ refers to the main coordinate, *f*_*u*_ and *f*_*v*_ respectively represents the focus length of the camera in horizontal and vertical direction. The direction angle *θ* of the feature point can be expressed finally as:

$$\theta =\arctan(\frac{d_{v}}{d_{u}})$$ (9)

#### Gravity improved FREAK description

The direction computing method of traditional FREAK descriptor is similar to BRISK descriptor. Both of them need to conduct the accumulated operation on the local gradient of the point pair selected at feature neighborhood. The gravity sensor on the mobile device not only can improve the calculating efficiency of feature extraction but also can solve the mismatching of feature points with physical similarities to some extent to improve the accuracy of feature matching.

**Fig 8:**
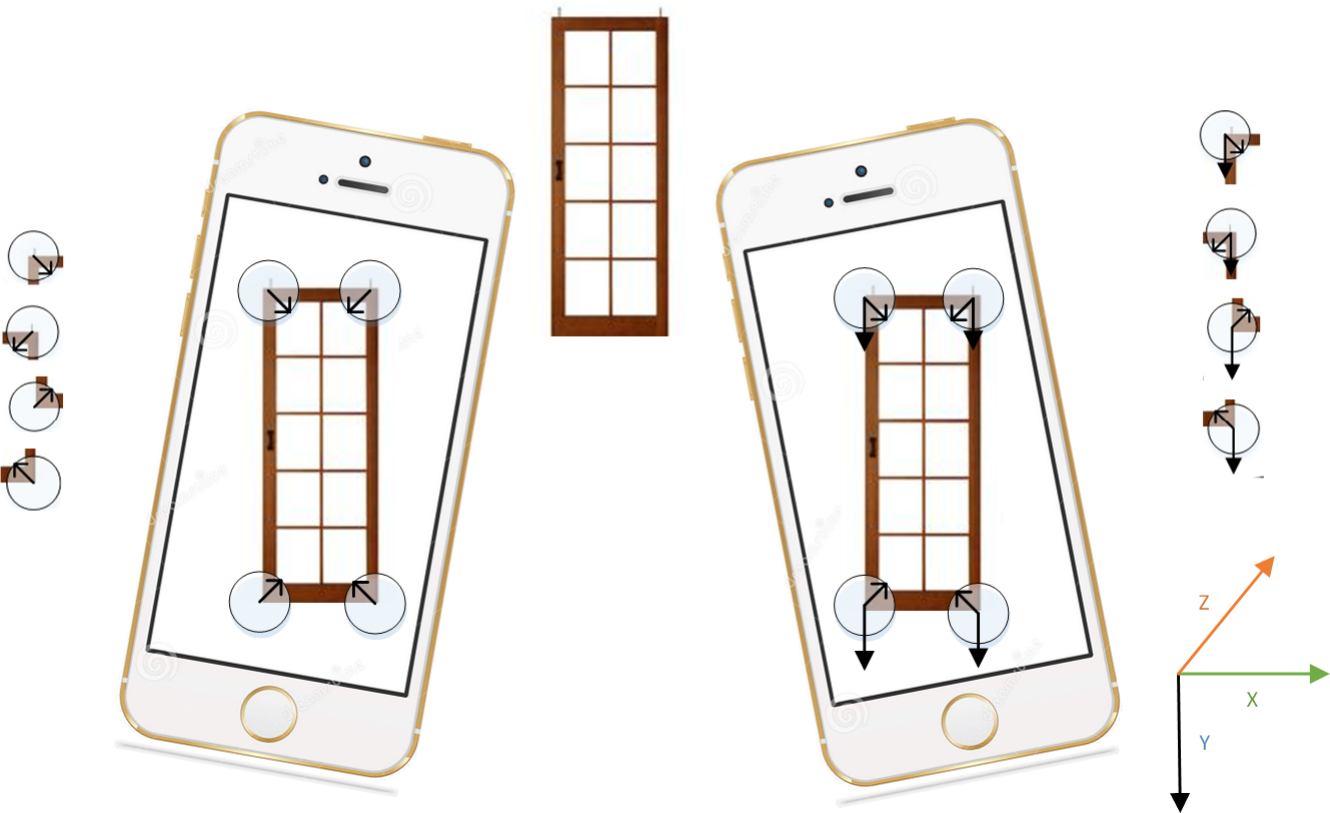
Feature point aligned in gray gradient direction (left) and gravity direction (right).

Take the condition described in the Fig 8 [34] as example, if the angular point in the image is described by traditional FREAK algorithm, it would rotate the feature point to current dominant gradient orientation during the normalization process. Although the feature points of the four window corners respectively represented four different physical points in reality scene, their descriptors is basically the same and the feature points are unable to be distinguished according to the descriptors. However, the gravity improved FREAK description algorithm has a completely different effect. For the four feature points in Fig 8, their feature directions are all straight down gravity directions from the window. During the normalization, each feature point will be rotated to the gravity direction as shown in Fig 8 (right). Obviously they have strong discriminative characteristics. Hence, the generated feature description vectors will be also different.

The theory of taking gravity direction as the feature direction can be understood as: when users are experiencing mobile augmented reality system, they will proceed with the augmentation with smartphone aligned with nearly vertical scene objective. First, phone camera will shoot the first frame of the scene image and the device sensor will record the present gravity and its direction g1. That is the phone describes the feature point in the first frame of picture along with the gravity components of x, y, z axles in Fig 7 with its projection of g1 on the image; due to the rotation of phone, the projection of g2 and g1 on the image of the gravity vector g2 pointed in different directions when shooting the second frame of image. So the second frame of image can be rotated until g2 and g1 have the same direction, which means the gesture and angle of the phone shooting the first frame of image was restored so that to maintain the rotation invariance of the feature point. Based on the above theory analysis, traditional FREAK description algorithm can be improved by using gravity angle instead of traditional feature point angle to calculate descriptor.

Compared with original FREAK algorithm, improved FREAK algorithm can accomplish the assignment of all the feature point directions in the same image only by using the build-in sensing data interface of mobile platform system once, which greatly improved the description efficiency of the feature point. In addition, due to the accuracy requirement of the sensor itself, the feature direction information obtained by gravity sensor is more accurate than that obtained by computer vision method, which can help feature point accomplish more sophisticated matching. Meanwhile, the experiment showed that traditional FREAK algorithm can preferably maintain the rotation invariance of feature point when the mobile device rotating around Z axle in Fig 7. The matching accuracy will be greatly decreased when the device rotates around other axles. While improved FREAK algorithm can better adapt the multi-directional rotating shooting and is more flexible.

## Mobile AR image matching and tracking registration

In the mobile augmented reality system, the obtaining of the feature point successfully matched in referential image and target image showed that homograph relation can be created between the two images and the registration of the augmented information can be accomplished. However, it is hard to avoid the mismatching in the real situation. Mismatching feature would affect the accuracy of 3D registration tremendously. Meanwhile, if every frame of image in the scene video is to be conducted with feature extraction and matching, it will consume the limited system resource on the mobile platform and affect the real-time characteristics of the mobile augmented reality system. Hence, to deal with the mismatching problem, RANSAC algorithm is used to eliminate exterior points and improve the final matching accuracy; meanwhile, the inherent continuity of video image will be fully excavated and the tracking registration of the video will be conducted through the improved KL algorithm based on inertial sensor to assure the fluency of mobile augmented reality system.

### Matching purification

In the image field, it is common to face the problem of estimating the image transformation matrix with matching feature points. How to obtain stable model parameter is the key point of conducting the matrix estimation. However, in the real feature matching, it is hard to assure the accuracy of all the matching data. Hence, it is of high priority to solve the problem of estimating correct model parameters by using the samples containing abnormal data.

#### Random sample consensus matching algorithm

First proposed by Fischler et al., random sample consensus matching algorithm (RANSAC) is an optimized algorithm used to estimate stable model parameters [26]. Based on RANSC algorithm, Chum et al. [24] proposed local optimization in 2003 to improve RANSAC algorithm and increase efficiency through accelerating the algorithm convergence. In 2004, Matas and Churn et al. [25] proposed the pre-testing model of *T*_*d*,*d*_, the speed of which has been improved 50% compared with typical RANSAC algorithm. On this basis, Chen and Wang[27] raised the pre-testing model of *T*_*c*,*d*_ in 2005, which is more generalized. After conducted a number of experiments, Capel [28] proved in the articles that the adding of local optimization and pre-testing model of RANSAC algorithm can also maintain the previous accuracy and reliability. Considering the limited resource of the mobile platform, this paper chose to use the PERANSAC algorithm proposed by Chen et al. to improve the overall efficiency [27].

#### Pre-testing rapid random sample consensus matching algorithm

PERANSAC algorithm[27] increased a pre-testing model based on RANSAC algorithm. First, part of sample data was chosen to evaluate current model parameters. Only those models that have passed the pre-testing are supposed to be used for the evaluation of all the rest samples. Besides, in order to maintain the algorithm reliability, PERANSAC increased the sampling data. The specific algorithm procedure is shown as follows:

Given the testing data number n, sample abnormal rate *ε* and minimum pre-testing pass rate, calculate *n*_*f*_ and *P*_*f*_ according to Formula 10; $C_{n}^{i}$ refers to the combination of i samples selected, *P*_*f*_ represents the test pass rate, *n*_*f*_ stands for the domain point number needed to pass the test;

$$1-\sum_{i=0}^{n_{f}-1}C_{n}^{i}\varepsilon^{\left(n-i\right)}{\left(1-\varepsilon \right)}^{i}=P_{f}$$ (10)

According to Formula 11, select the sample number M needed for the model parameter evaluation, m represents the minimum sample number needed for the model estimation;

$$1-{\left(1-{\left(1-\varepsilon \right)}^{m}P_{f}\right)}^{M}=P$$ (11)

1. Select a group of random samples and estimate the model parameters that the samples satisfy;
2. Select n random data, test the model parameters obtained from (3), and loop perform (3) (4) until all the parameters were tested. If all the parameters failed in the test, restart from (1). If the obtained model parameters still can’t pass the pre-test after many times of repeated operation, increase the sample abnormal rate *ε* and estimate again;
3. Estimate the succeeded models with all the sample data and write down the corresponding number of interior points;
4. Select the best model parameters under the standard of interior points number and error variance;
5. Based on the interior points included in the selected model parameters, estimate the model parameter that is the final results of the algorithm.

### Gravity-KLT improved tracking registration algorithm based on MEMS sensor

#### Kanade-Lucas Tracking algorithm

KL(Kanade-Lucas)Tracking algorithm uses window image registration technology for the tracking of feature. First proposed by Lucas.B.D et al. [29] and improved by Baker.S and Matthews.I[30], this algorithm has been a typical algorithm in the target tracking field. The early Lucas-Kanade algorithm aimed to realize the registration of template image *T*(***x***) and input image *T*(***x***), where ***x*** = (*x*,*y*)^*T*^ is the 2D column vector representing the pixel coordinates. Specific to optical flow computation, template *T*(***x***) represents the subdomain of the image at the time of t (e.g. image window); *I*(***x***) refers to the image at the time of t+1. If *W*(***x***,***p***) stands for the parameterized set of all reasonable deformation of the image, where ***p*** = (*p*_1_,…,*p*_*n*_)^*T*^ refers to the parameter vector. Then the parameterized set can map the pixel x in the coordinate system of template image T onto the *W*(***x***,***p***) position in the coordinate system of the image I. Taking the optical tracking method for example, *W*(***x***,***p***) can be expressed as:

$$W(x,p)=\left(\begin{array}{l} x+p_{1}\\ y+p_{2} \end{array}\right)$$ (12)

The vector parameter ***p*** = (*p*_1_,*p*_2_)^*T*^ refers to the optical flow vector. If the target to be tracked is an image block which can move freely in the 3D space, then *W*(***x***,***p***) can be assumed to represent affine transformation, that is:

$$\begin{array}{l} W(x,p)=\left(\begin{array}{l} (1+p_{1})\cdot x+p_{3}\cdot y+p_{5}\\ p_{2}\cdot x+(1+p_{4})\cdot y+p_{6} \end{array}\right)\\ \;=\left(\begin{array}{l} 1+p_{1}\;p_{3}\;p_{5}\\ p_{2}\;1+p_{4}\;p_{6} \end{array}\right)\left(\begin{array}{l} x\\ y\\ 1 \end{array}\right) \end{array}$$ (13)

where ***p*** = (*p*_1_,*p*_2_,*p*_3_,*p*_4_,*p*_5_,*p*_6_)^*T*^ denotes Vector parameter. Obviously, under different tracking environment, *W*(***x***,***p***) can be any complicated target tracking template with variable dimension of the parameter vector.

Essentially, the ultimate goal of Lucas-Kanade algorithm is to reduce the mean-square error between the template image T and the transformed image I to the most:

$$\sum_{x}{\left[I(W(x;p))-T(x)\right]}^{2}$$ (14)

As shown in Formula 14, tracking problem was transformed to a nonlinear optimization problem so that to calculate the vector p to satisfy the target of the algorithm. In order to obtain the best solution of Formula 14, Lucas-Kanade algorithm assumes the estimation of p is known, and calculate Δ***p*** and conduct the iterative updates of p until its convergence, as shown in Formula 15 and Formula 16:

$$\sum_{x}{\left[I(W(x;p+\Delta p))-T(x)\right]}^{2}$$ (15)

p←p+Δp (16)

Whether p is convergent or not is usually decided when the template of the vector Δ***p*** is lower than a threshold value.

In the Δ***p*** computing process, in order to linearize Formulas 15, *I*(*W*(***x***;***p*** + Δ***p***)) will be conducted with Grade 1 Taylor Series unfolding and Formulas 17 can be obtained:

$$\sum_{x}{\left[I(W(x;p))+\nabla I\frac{\partial W}{\partial p}\Delta p-T(x)\right]}^{2}$$ (17)

where $\nabla I=(\frac{\partial I}{\partial x},\frac{\partial I}{\partial y})$ refers to the grade value of image I at *W*(***x***;***p***). Normally, ∇*I* will be calculated first at the coordinates of image I and then mapped to the *W*(***x***;***p***) position of template image T. $\frac{\partial W}{\partial p}$ is the Jacobian determinant of *W*(***x***;***p***). If *W*(***x***;***p***) = (*W*_*x*_(***x***;***p***),*W*_*y*_(***x***;***p***))^*T*^, then:

$$\frac{\partial W}{\partial p}=\left(\begin{array}{l} \frac{\partial W_{x}}{\partial p_{1}},\frac{\partial W_{x}}{\partial p_{2}}\ldots \frac{\partial W_{x}}{\partial p_{n}}\\ \frac{\partial W_{y}}{\partial p_{1}},\frac{\partial W_{y}}{\partial p_{2}}\ldots \frac{\partial W_{y}}{\partial p_{n}} \end{array}\right)$$ (18)

Traditionally, the result of column vector calculating partial derivatives can be described by vector. The chain rule can change to matrix multiplication for calculation and the Jacobian determinants of Formula 13 can be:

$$\frac{\partial W}{\partial p}=\left(\begin{array}{llllll} x & 0 & y & 0 & 1 & 0\\ 0 & x & 0 & y & 0 & 1 \end{array}\right)$$ (19)

If the partial derivative related to Δ***p*** is calculated on the basis of Formula 17:

$$2\sum_{x}{\left[\nabla I\frac{\partial W}{\partial p}\right]}^{T}[I(W(x;p))+\nabla I\frac{\partial W}{\partial p}\Delta p-T(x)]$$ (20)

$\nabla I\frac{\partial W}{\partial p}$ is regarded as the steepest gradient descent image. If Formula 20 = 0, then:

$$\Delta p=H^{-1}\sum_{x}{\left[\nabla I\frac{\partial W}{\partial p}\right]}^{T}[T(x)-I(W(x;p))]$$ (21)

H refers to the Hessian matrix of:

$$H=\sum_{x}{\left[\nabla I\frac{\partial W}{\partial p}\right]}^{T}[\nabla I\frac{\partial W}{\partial p}]$$ (22)

If $\sum_{x}{\left[\nabla I\frac{\partial W}{\partial p}\right]}^{T}[T(x)-I(W(x;p))]$ is considered as the steepest gradient descent parameter update, then Formula 22 means Δ*p* is actually the multiplication of steepest gradient descent parameter update and Hessian matrix inverse matrix. Therefore, Lucas-Kanade algorithm can repeat the iteration of Formula 16 and Formula 17 until convergence occurs or exceeds the iteration times. The specified procedure is shown follows:

1. Calculate the transformation *I*(*W*(***x***;***p***)) of image I according to current parameter p;
2. Calculate the error image *T*(*x*) − *I*(*W*(***x***;***p***));
3. Use *W*(***x***;***p***) to transform the gradient image ∇*I*;
4. Estimate the Jacobian determinants $\frac{\partial W}{\partial p}$ at position (***x***;***p***);
5. Calculate the steepest gradient descent image $\nabla I\frac{\partial W}{\partial p}$;
6. Calculate Huessian matrix according to Formula 22;
7. Calculate the steepest gradient descent parameter update $\sum_{x}{\left[\nabla I\frac{\partial W}{\partial p}\right]}^{T}[T(x)-I(W(x;p))]$;
8. Calculate Δ*p* according to Formula 21;
9. Update parameter p according to the Formula 16;

Until ‖Δ*p*‖ ≤ *ε*

After analyzing the procedure of the above algorithm, as parameter vector p changes with the increase of iteration, steps from (1) to (9) need to be recalculated at each iteration. In order to reduce the time complexity of algorithm, some scholars proposed to use the method of reverse combination to conduct the parameter update. The main idea of the method is to exchange the roles of image I and template T, and use current deformation matrix and reverse matrix of augmented matrix to compose a new deformation matrix so that to replace the method of updating through incremental parameter vector p as shown in Formulas 23 and Formulas 24:

$$\sum_{x}{\left[T(W(x;\Delta p))-I(W(x;p))\right]}^{2}$$ (23)

$$W(x;p)\leftarrow W(x;p)\circ W{\left(x;\Delta p\right)}^{-1}$$ (24)

Unfold the Formulas 23 at Level I Taylor series to obtain Formula 25:

$$\sum_{x}{\left[T(W(x;0))+\nabla T\frac{\partial W}{\partial p}\Delta p-I(W(x;p))\right]}^{2}$$ (25)

Without loss of generality, *W*(*x*;0) can be assumed as the identical transformation, saying *W*(*x*;0) = *x*. Then the solution of the minimum square problem is:

$$\Delta p=H^{-1}\sum_{x}{\left[\nabla T\frac{\partial W}{\partial p}\right]}^{T}[I(W(x;p))-T(x)]$$ (26)

Where H refers to the Hessian matrix which replaced I with T:

$$H=\sum_{x}{\left[\nabla T\frac{\partial W}{\partial p}\right]}^{T}[\nabla T\frac{\partial W}{\partial p}]$$ (27)

As current Hessian matrix has nothing to do with parameter vector p, it is unnecessary to repeat the calculation at each of iteration but calculate once and store in advance, which improved the efficiency of the algorithm.

#### Improved KL algorithm based on MEMS sensor

There are many restrictions when applying traditional KL feature tracking algorithm. The algorithm assumes the constant target brightness and allows only small movements of the object in adjacent frames. The first restriction is to assure the mean square error of target image and template image not influenced by illumination, and the later part is to assure KL algorithm could find out the solution to satisfy the threshold condition. However, in the actual application of mobile augmented reality, it is common when the device needs to move in a large scale or rotate rapidly, which makes traditional KL algorithm unable to accurately track the rapid and large-scale moving target scenes.

Considering the regular application scenes of mobile augmented reality, this paper uses inertial sensor to help KL algorithm select parameters and takes the affine illumination model proposed by H.Jin et al. [31] as the tracking model of the algorithm. This model contains 8 parameters: *p* = (*a*_1_,…,*a*_6_,*α*,*β*). In the model, affine matrix (*A*,*b*) is used to describe the movement of the device in the space, such as translation and rotation; proportion and migration model (*α*,*β*) is used to deal with the light contrast change of the template image; Alpha proportion is used to compensate the change of environment light and Beta migration is used to compensate the change of direct light. The whole model can be expressed as:

T(x;p)=(α+1)T(Ax+b)+β (28)

In which

$$A=\left[\begin{array}{l} 1+a_{1}\;a_{2}\\ a_{3}\;1+a_{4} \end{array}\right],b=\left[\begin{array}{l} a_{5}\\ a_{6} \end{array}\right]$$ (29)

**Fig 9:**
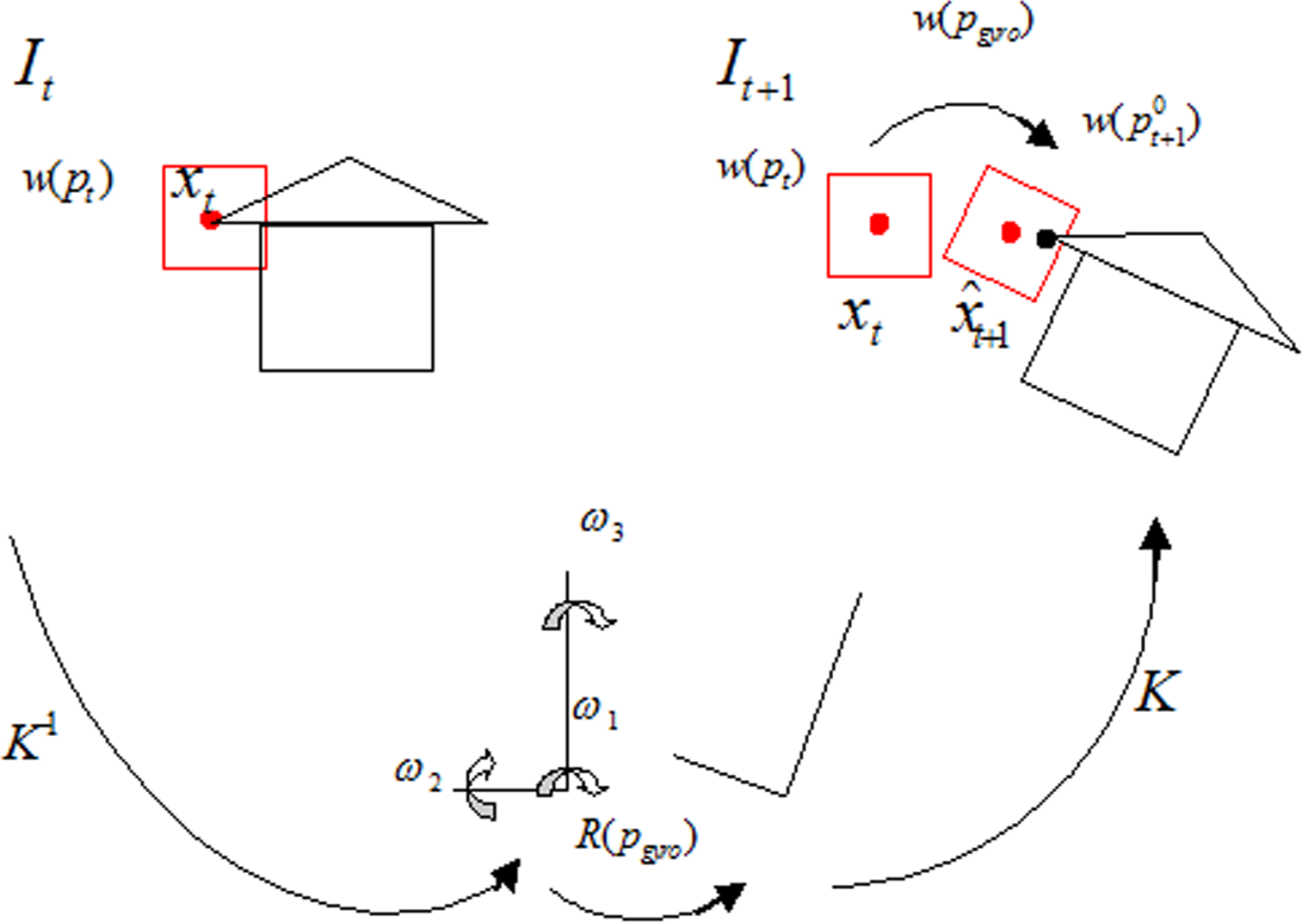
Diagram of KL tracking algorithm based on inertia.

During the tracking progress, most of the introduction of light stream comes from the change of camera posture. Hence, three-axis gyroscope sensor can be used to record the instantaneous rotation $q_{t}^{t+1}$ of current camera and $p_{t+1}^{0}$ can be predicted according to the current deformation parameter vector *p*_*t*_ as shown in Fig 9.

Suppose the difference between image *I*_*t*_ and image *I*_*t*+1_ is caused by the rotation $R_{t}^{t+1}$ of camera, then under the premise of given the calibration parameter matrix K of camera, all the movements of feature points can be described by a 2D Homography matrix H:

$$H=K^{-1}R_{t}^{t+1}K$$ (30)

Homography H is more advanced than the affine transformation (*A*,*b*) used for tracking target change in the model. So (*A*,*b*) can be obtained directly from Homography matrix. First, homography matrix needs to be standardized to be *H*_3×3_. The linear transformation part *A*_*pred*_ is the same as upside submatrix of matrix *H*_3×3_, describing the affine parts as rotation and dimension, etc. Essentially, *b*_*pred*_ is the position offset caused by 2D homography matrix such as the position offset *x*_*H*_ ≡ *H*[*x* 1]^*T*^ generated by point x through H. The illumination parameters in the model will not be influenced, so:

$$\left\{ \begin{array}{l} A_{pred}=H_{2\times 2}\\ b_{pred}\;=x_{H}-x\\ \alpha_{pred}\;=\beta_{pred}=0 \end{array}\right.$$ (31)

In conclusion, the initial parameter of image *I*_*t*+1_ can be obtained through the forward combination of sensor prediction parameter *p*_*pred*_ and current parameter *p*_*t*_, that is:

$$\begin{array}{l} W(x;p_{t+1}^{0})=W(x;p_{pred})\circ W(x;p_{t})\\ \;=W(W(x;p_{pred}),p_{t}) \end{array}$$ (32)

Guided by the test data from the sensor, $p_{t+1}^{0}$ is more easily to drop in the convergence interval C compared with *p*_*t*_.

## Design and realization of mobile augmented reality system

In the experiments, MEMS sensor data is applied to realize FREAK feature extraction and to improve algorithms as KL tracking registration.

### System environment

Since iPhone is selected to perform the establishment process of the mobile augmented reality system in this study, the development tasks are mainly finished in MacOS X 10.9 Mavericks. The main body of the system is programmed with Objective-C an object-oriented extension of C Programming Language with perfect downward compatibility with the existing image algorithm’ C Language Library as OpenCV. The system project is compiled and published with Apple XCode 4.5, and then finally operated in iPhone5.

### System process and structure

**Fig 10:**
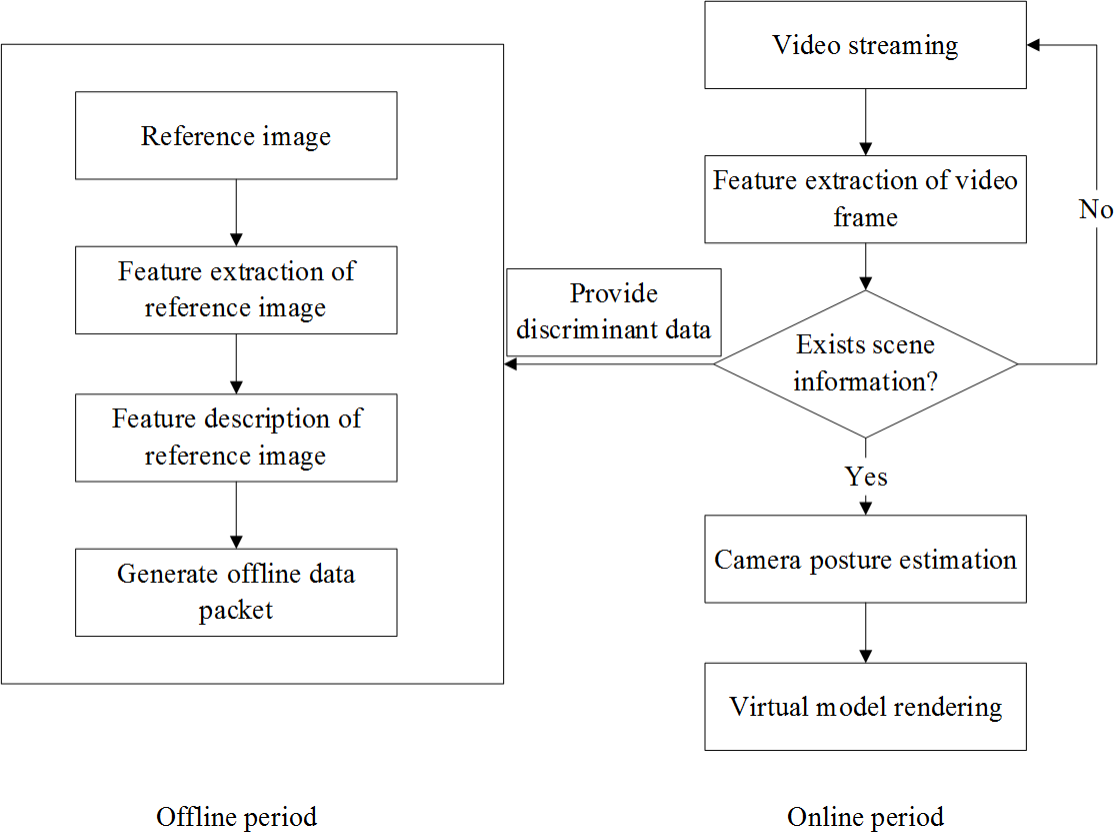
Flowchart of mobile augmented reality system.

As shown in Fig 10, the system presented in this paper is mainly composed of two parts, namely offline stage and online stage. In offline stage, feature extraction and description are conducted for the reference image to form a feature data package, which will be applied as a matching basis in online stage.

In offline stage, every reference image will be described in two different ways, which are the traditional FREAK description algorithm and the gravity-based FREAK description algorithm. Since these procedures can all be completed in PC offline, they would not affect the registration efficiency of the mobile terminal.

The online procedures are the acquisition of video stream, the judgment of scene information, the estimation of camera pose and the display of model rendering, which are mainly finished on iPhones. Before the follow-up procedures, the video frame obtained with iPhone5 HD camera should be adjusted to the size of 480x640 to reducing the computation complexity in iPhone5.

**Fig 11:**
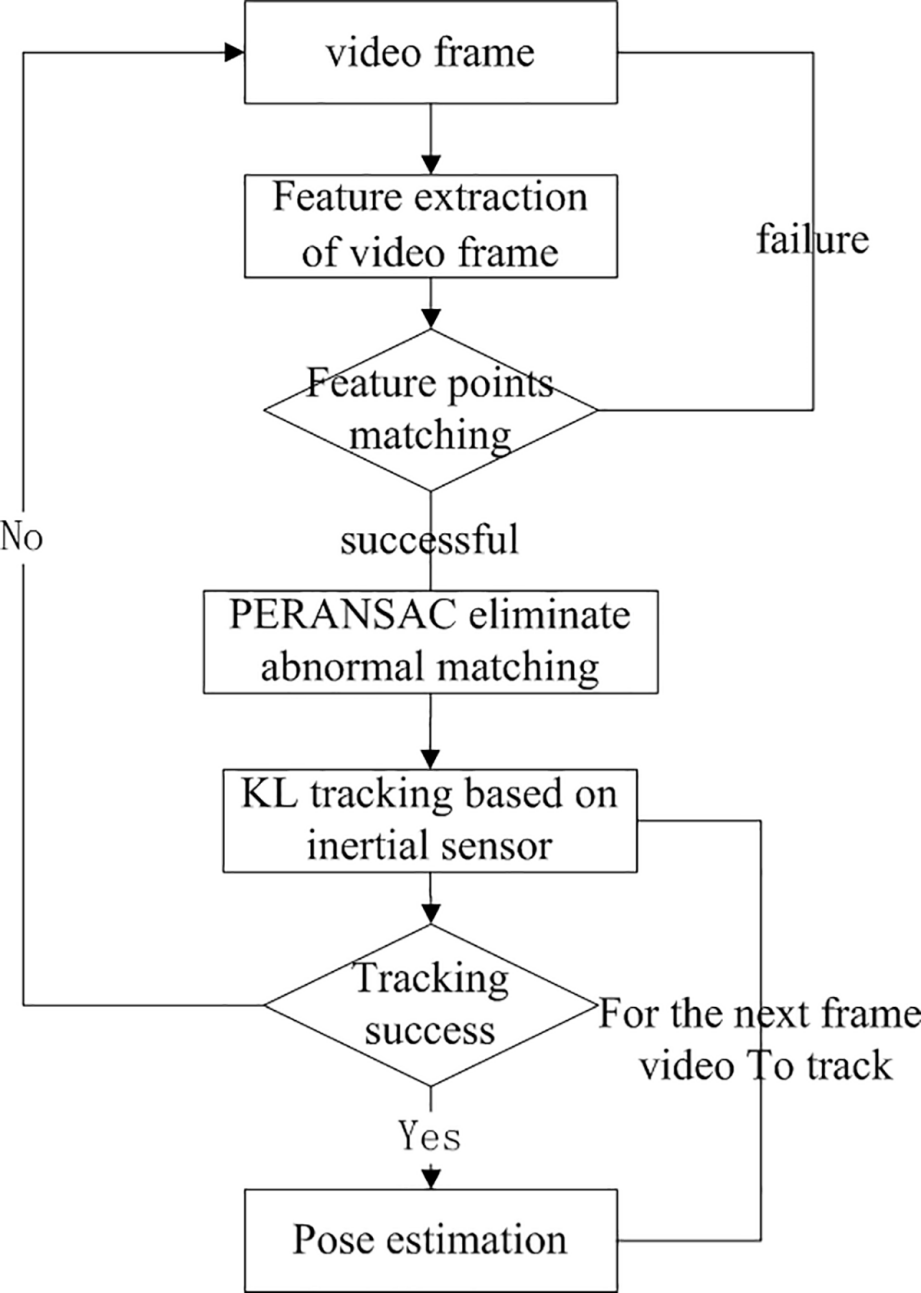
Flowchart of scene detection and registration tracking.

The process of scene information is the core of the entire registration flow, which includes the matching and tracking of video features. Detailed procedures are shown in Fig 11.

First, obtain a frame of video image and extract its feature description. On augmented or approximate horizontal planes, mobile phones cannot produce significant project-vector on imaging plane due to gravity factor. In this case, the system can shift to the traditional FREAK description algorithm and use the gray level of feature points’ adjacent area to set the feature direction. This is also the reason why two algorithms are required in offline stage.

Then, change the algorithm according to the current pose of the mobile phone estimated by the built-in MEMS sensor of iPhone. If it similar to vertical pose, Gravity-FREAK algorithm will be applied to conduct the feature point description, otherwise FREAK algorithm will be selected.

Match the obtained feature points with those of the reference image and use PERANSAC algorithm to screen the matching features. Remove the abnormal data that cannot meet the requirements. If the rest matching feature is larger than a certain threshold value (e.g. 20), it indicates that the objective scene do exist in this image frame. In this case, the rest matching points can be used to evaluate the projection matrix between the reference image and the current image.

Meanwhile, in order to make full use of the continuity of two adjacent video frames, the projection matrix from the reference image to image t will be recorded when the objective scene is detected in a certain image frame t. Gravity-KL will then utilized to track the scene. If the scene is successfully traced, the transition matrix of this image frame relative to the former image frame can also be obtained; iterate it to image t to calculate the projection matrix from the reference image to this image frame, which marks the completion of the track registration. If the track failed, return to the scene detection stage and restart the feature matching. Change the projection of the virtual model in line with the projection matrix from the reference image to the current video image; render and superimpose it to the right position in the current image to acquire a fusion image, thus realizing the augmentation of the reality scene. The tree-dimensional model used in this paper is created with 3dxMax and rendered with OpenGL after format conversion.

### Experimental results and analysis of the MEMS-based Gravity-FREAK algorithm

The tool platforms applied to establish the experimental environment are listed in Table 1.

**Table 1:** Introduction of experimental environment.

Development Environment	Mac mini(OS X Mountain Lion), Xcode 4.5, OpenCV for iOS 2.4.2
Experimental Environment	iPhone5
Experimental Database	AR Database provided by Metaio Company, real scenes photographed by the authors

**Fig 12:**
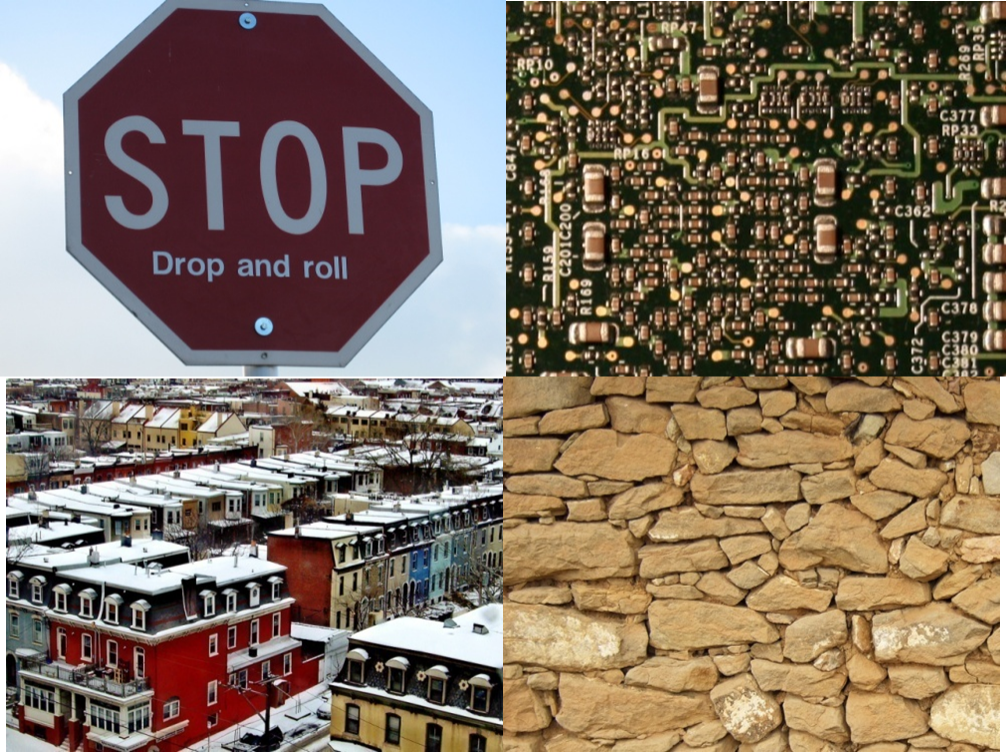
Reference images in Lieberknecht, S.et al.(2009).

The AR Database used in this study was proposed by Lieberknecht, S.et al. 2009 [32]. As shown in Fig 12, there are totally four images in this database. The first one is an image of a stop sign, which is relatively monotonous and with a few texture features (referred to as Stop Sign); the second image is about a Mac Mini Board with many repetitive and similar physical structures (referred to as Board); the third image describes a part of residential area in Philadelphia, representing typical outdoor scenes (referred to as Philadelphia); the last image is the front view of a stone wall with relatively rich texture structures (referred to as Wall). Use smart phone to photograph these four images when they are placed in vertical planes; the resolution of the photos is set to be, which is convenient for the follow-up procedures. The experimental assessment of the algorithm performance consists of two aspects: the matching time feature description speed and accuracy had improved.

#### Time consumption assessment of the algorithm

**Fig 13:**
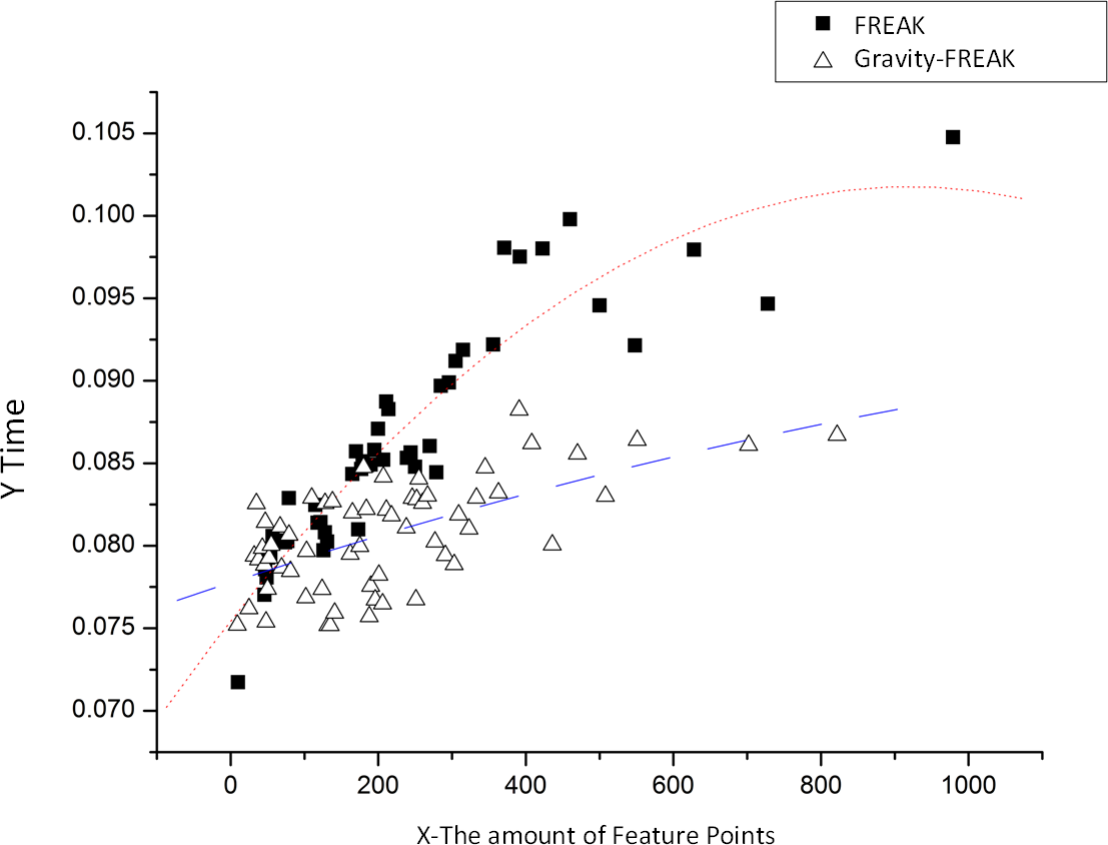
Tendency for time consumption of feature description algorithm to change with feature points numbers.

The first experiment only evaluates the feature descriptions. Since a single experiment may be influenced by errors, five repeated experiments are conducted in this study to calculate an average value to obtain the tendency (see Fig 13) for feature description algorithm’s time consumption to change with the number of feature points.

It is obvious that Gravity-FREAK algorithm has smaller cost, especially when the number of extracted feature points increases. The reason for this phenomenon is that Gravity-FREAK algorithm can carry out the evaluation of all the feature points in the direction of gravity projection with only one round of data acquisition through the sensor, while the traditional FREAK algorithm has to calculate the direction of each feature point separately. In general, Gravity-FREAK algorithm can save 10~20ms, which has great significance for the improvement of real-time property of the mobile augmented system.

#### Accuracy assessment of the algorithm

Under the condition that the reference images are placed on vertical planes, the mobile phone is rotate for different angles by the researchers in varied patterns. Meanwhile, the matching accuracy is recorded to serve as the evaluation standard of rotation non-deformation as shown in Formula 33.

$$Matching\;Accuracy=\frac{Correct\;Matching\;Feature\;Point\;Number}{Wrong\;Matching\;Number+Correct\;Matching\;Number}$$ (33)

For each objective image, four angular points are manually selected by the researchers to rapidly count the correct matching feature points and to evaluate the homographic matrix between this objective image and the reference image. Besides, this matrix is also applied to calculate the projection location of each matching feature point on the reference image. If the distance between the projection location and the matching location of the reference image is lower than a certain threshold value (e.g. 6 pixels), this feature point is determined as a correct matching feature point. In this way, the matching accuracy of each objective image can be quickly calculated. In addition, the mobile phone is also rotated along with the x, y and z axis (see Fig 7) respectively to evaluate the performance of the algorithm in different rotation patterns. Detailed experimental results are listed in Tables 2, 3 and 4.

**Table 2:** Comparison experiment of the traditional and improved FREAK algorithm as iPhone rotates along Z axis.

	Traditional FREAK Algorithm (%)				Improved FREAK Algorithm (%)			
	Stop Sign	Board	Philadelphia	Wall	Stop Sign	Board	Philadelphia	Wall
0	79.20	22.57	73.48	32.88	92.73	36.92	75.28	48.41
10	67.58	18.00	67.78	30.48	84.15	30.44	69.03	37.44
20	61.91	18.87	58.56	24.00	80.21	25.32	56.21	43.86
30	65.55	16.21	61.79	27.73	74.08	23.18	54.06	31.95
40	54.96	15.92	52.89	20.04	70.35	17.86	58.04	39.96
50	48.05	13.91	46.64	16.14	62.93	21.39	37.32	28.43
60	50.68	11.32	41.76	13.90	68.56	21.73	33.42	25.29
70	44.83	12.18	45.57	10.35	64.82	19.21	48.49	26.68
80	45.62	9.73	43.50	11.90	58.66	17.38	44.38	22.08
90	42.38	11.42	42.37	12.33	53.49	18.92	43.93	24.53

**Table 3:** Comparison experiment of the traditional and improved FREAK algorithm as iPhone rotates along Y axis.

	Traditional FREAK Algorithm (%)				Improved FREAK Algorithm (%)			
	Stop Sign	Board	Philadelphia	Wall	Stop Sign	Board	Philadelphia	Wall
0	79.20	22.57	73.48	32.88	92.73	36.92	75.28	48.41
10	70.91	19.60	62.58	25.48	88.03	27.48	65.93	42.93
20	56.37	14.64	63.34	19.91	74.37	21.21	67.92	35.41
30	57.48	10.13	51.29	13.78	70.73	16.35	58.43	34.44
40	46.74	11.91	40.79	15.57	63.65	19.80	53.59	28.82
50	32.43	9.93	35.66	11.66	52.91	13.69	51.16	22.61
60	31.57	6.74	31.25	8.07	56.44	11.84	52.20	15.17

**Table 4:** Comparison experiment of the traditional and improved FREAK algorithm as iPhone rotating along X axis.

	Traditional FREAK Algorithm (%)				Improved FREAK Algorithm (%)			
	Stop Sign	Board	Philadelphia	Wall	Stop Sign	Board	Philadelphia	Wall
0	79.20	22.57	73.48	32.88	92.73	36.92	75.28	48.41
10	78.16	20.97	68.02	24.93	85.72	28.06	71.54	43.79
20	67.05	17.50	54.48	20.72	81.02	23.89	65.13	38.27
30	60.50	19.41	43.42	17.92	73.41	17.97	58.84	26.89
40	48.96	15.84	41.65	19.28	66.45	18.68	40.96	30.50
50	42.32	14.12	36.89	13.13	54.84	15.31	44.78	24.25
60	37.66	10.20	25.31	15.86	65.83	16.83	47.27	22.20

**Fig 14:**
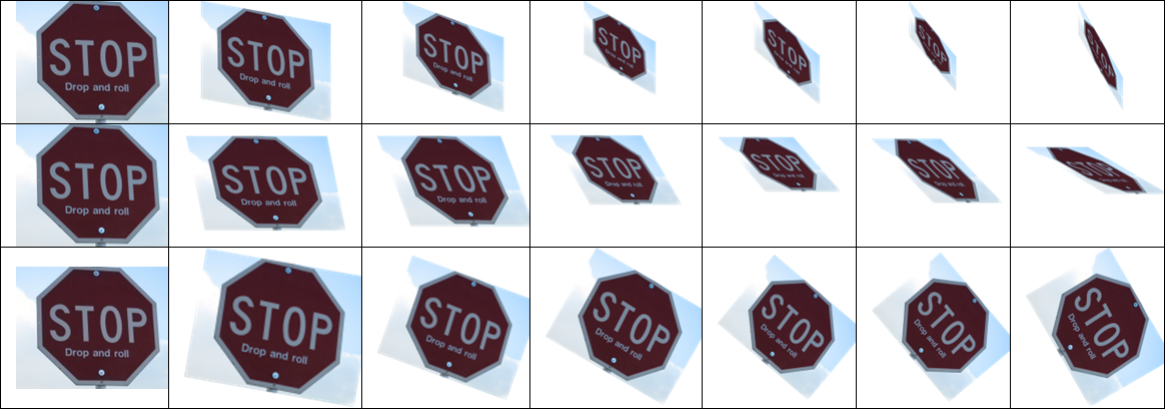
Deformation of the object as iPhone rotating along X, Y and Z Axis respectively.

In the above three tables, the left columns are about the matching accuracy of the traditional FREAK algorithm at different rotation angles, and the right columns are that of the improved FREAK algorithm. The comparison data of the matching accuracy when the mobile phone rotates along Z axis is list in Table 2. Pictures taken under this condition only rotate within the plane, and this is the most common rotation pattern (see the 3rd row of Fig 4). It can be concluded from the Table 2 that the improved FREAK algorithm has better accuracy than the traditional FREAK algorithm for in-plane rotation under the same conditions, and this is because the utilization of gravity as feature direction can exclude some wrong matching feature points of the neighborhood with similar grayscale (see Fig 14). Furthermore, the experiments have demonstrated that Gravity-FREAK algorithm also has great advantages over the traditional algorithm in complex scenes with many repeated textures (e.g. Board and Wall). The reason for this result is that Gravity-FREAK algorithm can better discriminate different features with similar textures since it applies the built-in gravity sensor of the mobile phone to screen the rotation directions of the feature points. Therefore, the theory presented in this section is feasible.

**Fig 15:**
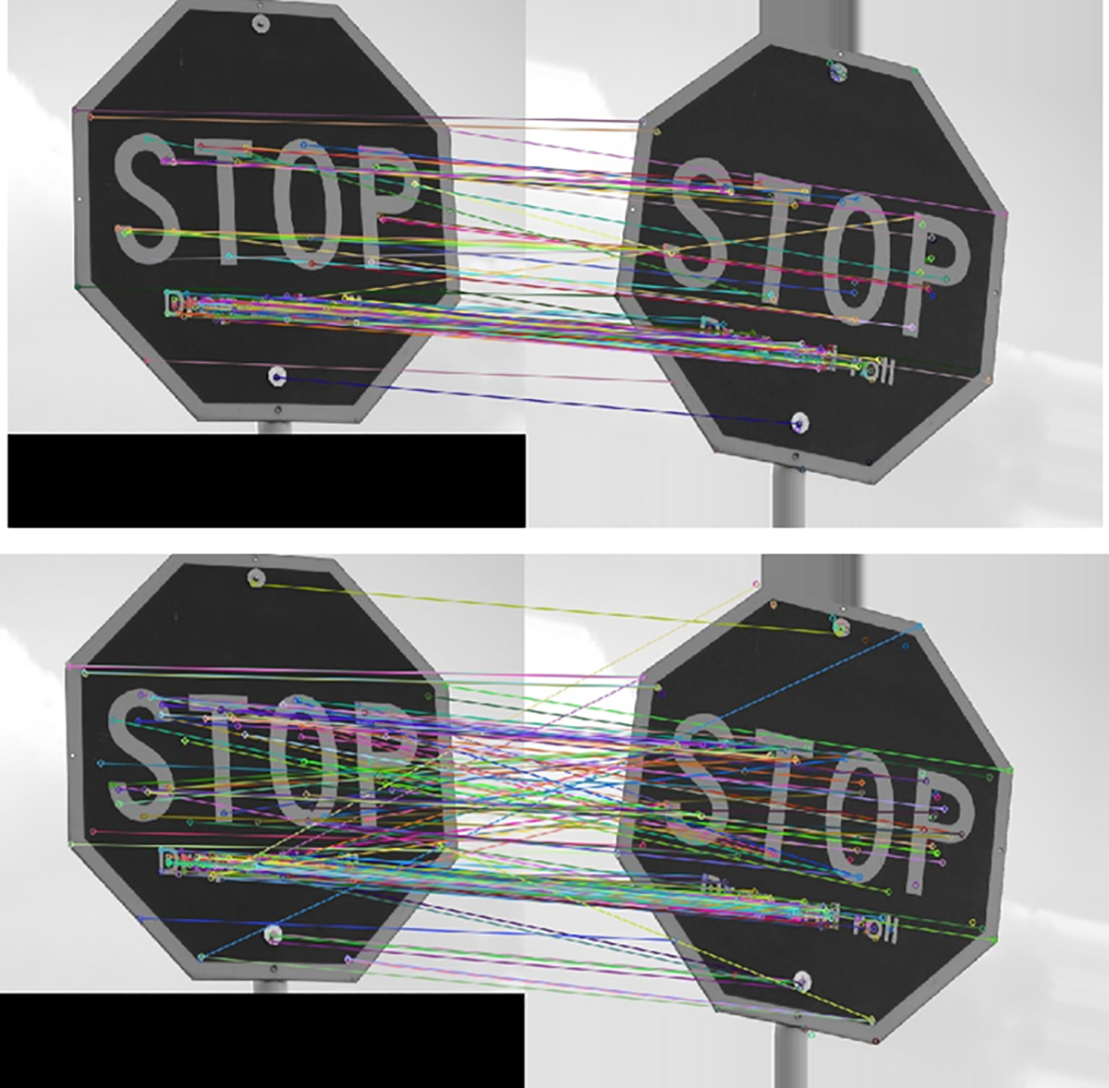
Comparison of matching accuracy of the improved (above) and traditional (below) FREAK algorithm.

The matching accuracy of different algorithms when the mobile phone is rotated in non-planar conditions (see the 1st and 2nd row of Fig 15) is listed in Tables 3 and 4. If the non-planar angle is too large, the objective image will have no practical significance. Hence, only experimental results of non-planar rotations within 0~60° are listed in this paper. The findings reveal that the matching accuracy will decrease with the traditional FREAK algorithm in non-planar rotations, while the Gravity-FREAK algorithm presents significant adaptability.

**Fig 16:**
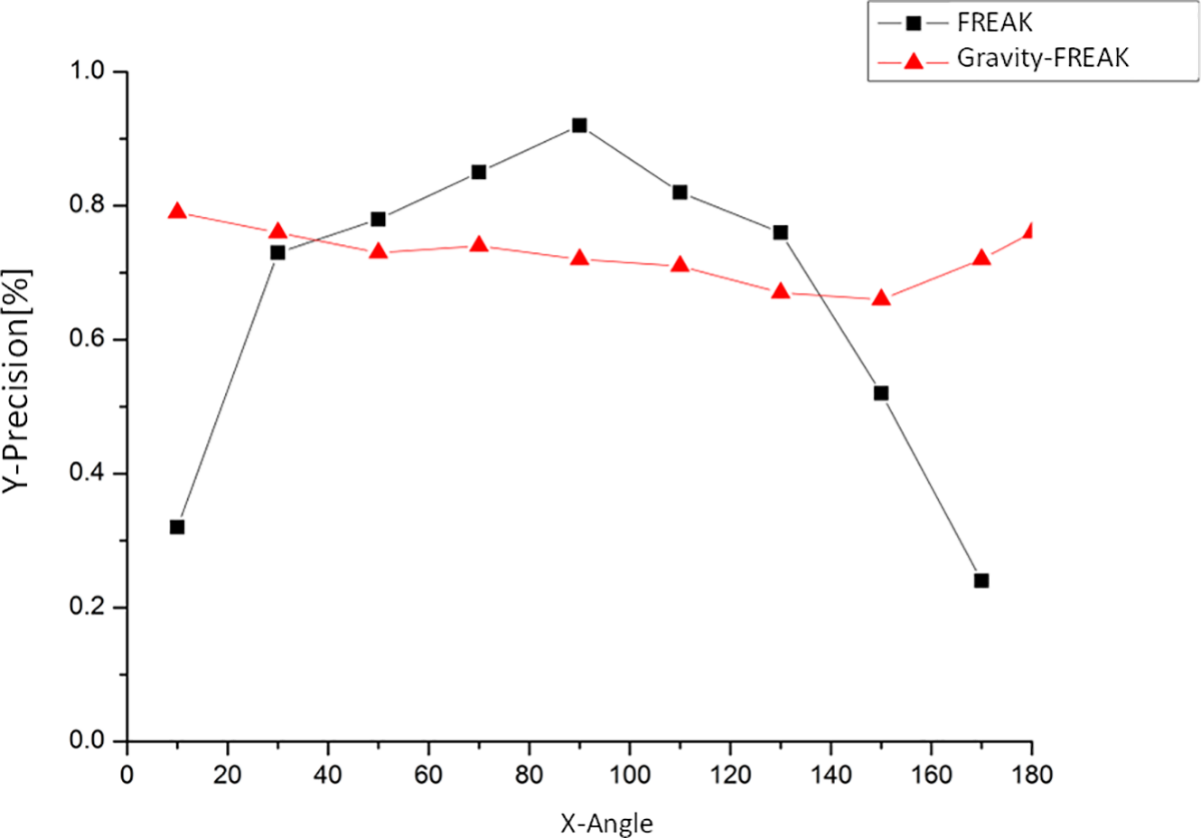
Diagram of algorithm performance varying with positional plane angles.

Additionally, the applicable environment of the algorithm is also evaluated. The detailed data of algorithm matching accuracy is obtained by changing the angles of the positional planes when photographing the Stop Sign (see Fig 16).

As shown in Fig 16, the best performance is obtained with Gravity-FREAK algorithm. Moreover, Gravity-FREAK algorithm can generate better accuracy than the traditional algorithm in a certain range (40~130). In extreme conditions, namely, the positional plane parallels to the horizontal plane, the Gravity-FREAK algorithm becomes invalid since it cannot acquire gravity projection, and this problem will be overcome in future studies. The solution to this problem will be of great practical significance since there are many vertical or approximate vertical objectives in real environment.

### Experimental for gravity-KLT algorithm

In this study, iPhone5 is chosen as the carrier of the experimental program; scene videos in the size of 640X480 resolutions and the frequency of 30Hz are applied as the input source to test the tracking results of the original and improved KL algorithms. The rotation movements of iPhone5 along X, Y and Z axis are recorded in the scene video, and the current rotation parameters are also recorded with the gyroscope for mobile phone.

**Fig 17:**
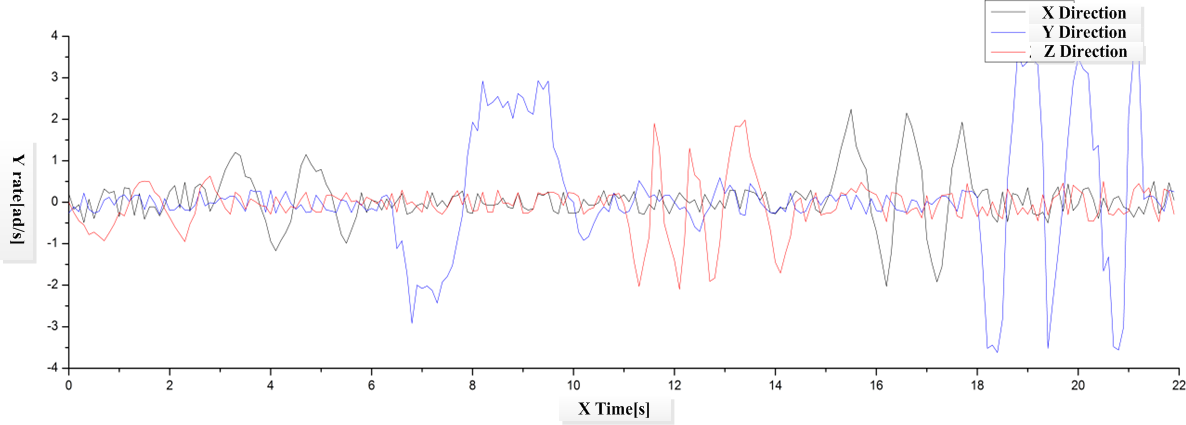
Diagram of iPhone5 angular velocity change.

**Fig 18:**
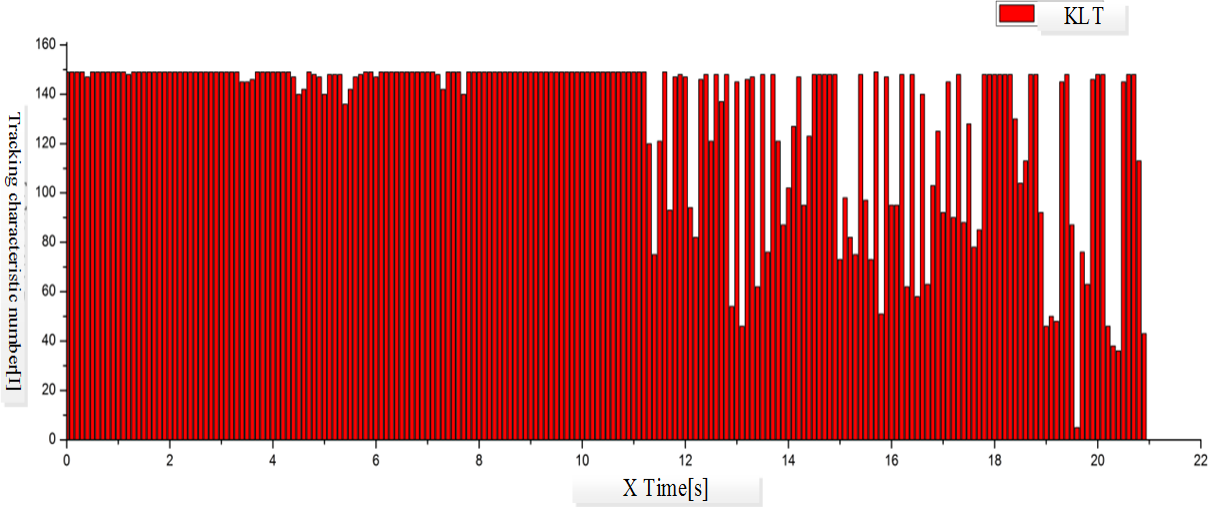
Diagram of tracking feature change of traditional KLT algorithm.

**Fig 19:**
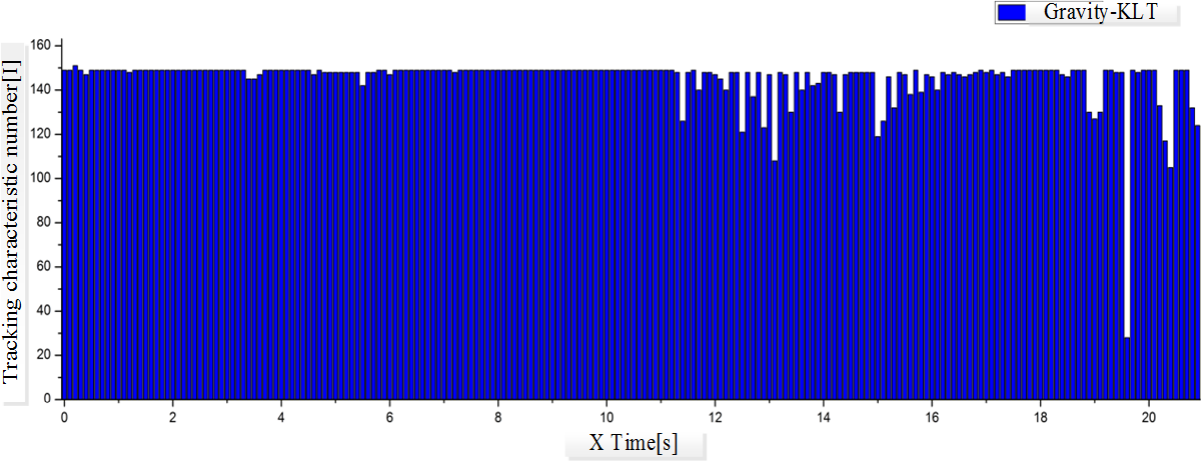
Diagram of tracking feature change of improve KLT algorithm.

**Fig 20:**
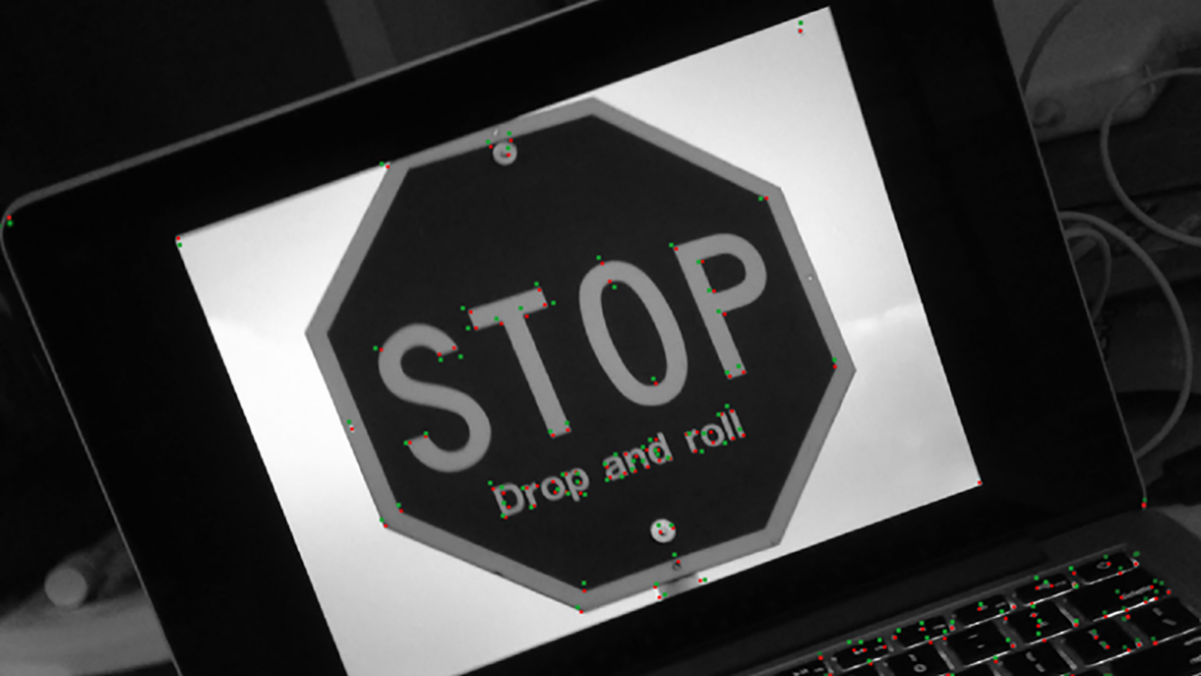
Prediction points (Green) achieved with improve KL algorithm and traced feature points (Red) of former frame.

In the scene video as illustrated in Fig 17, the mobile phone firstly rotates along X, Y and Z axis separately at low speed (0~11s), and then at high speed (12~22s). According to Fig 18 and Fig 19, both algorithms have achieved good tracking results in slow movements. When the mobile phone starts to rotate at high speed, the traditional KLT algorithm presents big fluctuation as the angular velocity changing violently. It indicates that the traditional KLT algorithm fails to acquire the tracking features in rapid movements, which would further result in the loss of targets. On the contrary, the improve algorithm can predict the positions of feature points with the rotation parameters obtained through the sensor, and then starts the search from these positions. As shown in Fig 20, this kind of prediction is quite precise due to the support of the measured data of the sensor. In other words, the improved KLT algorithm can achieve good tracking effects when the mobile phone is rotating at high speed.

### System display and analysis

**Fig 21:**
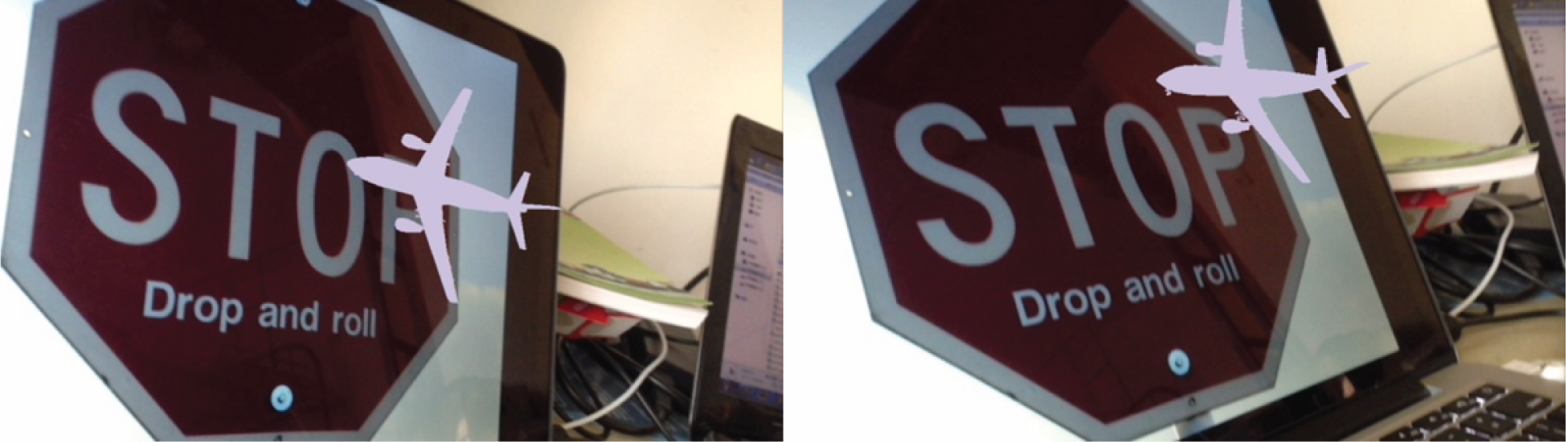
The augmented effect of the system when the phone rotates around X axle.

**Fig 22:**
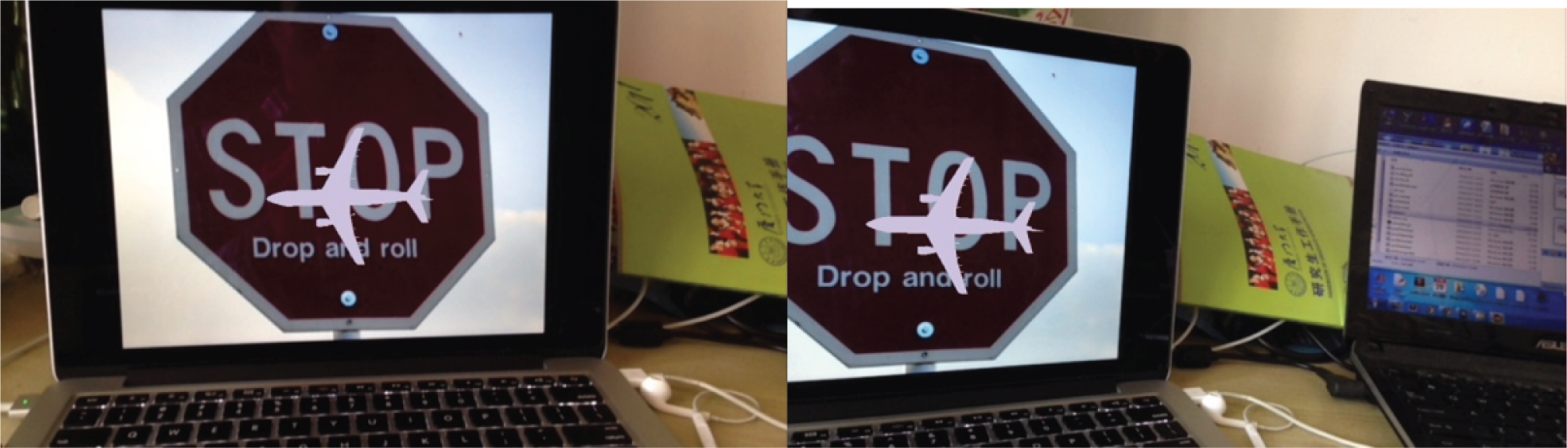
The augmented effect of the system when the phone rotates around Y axle.

**Fig 23:**
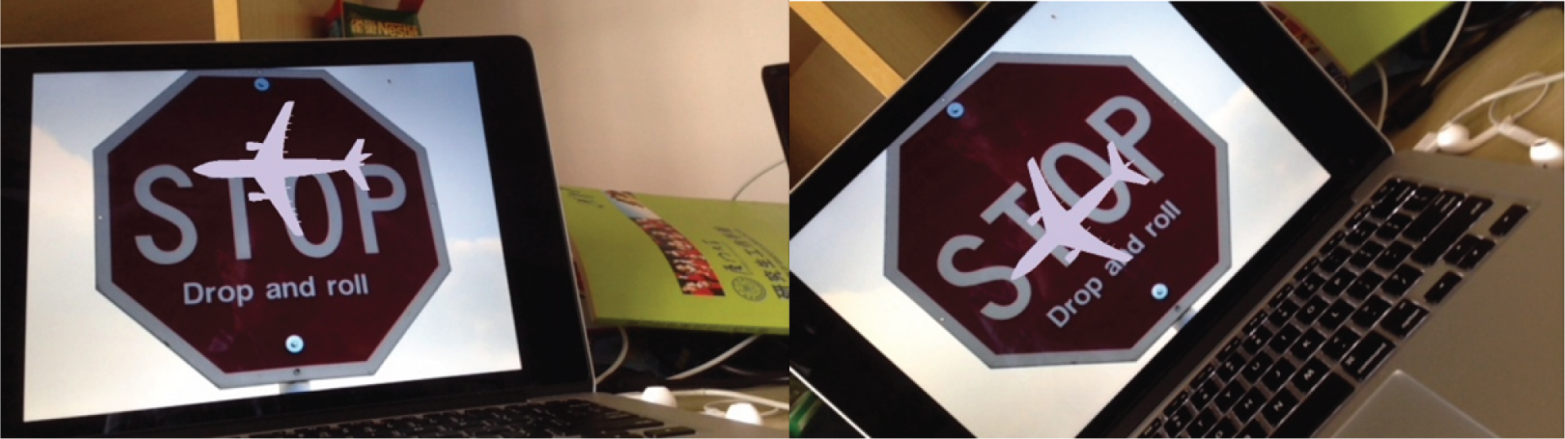
The augmented effect of the system when the phone rotates around Z axle.

**Fig 24:**
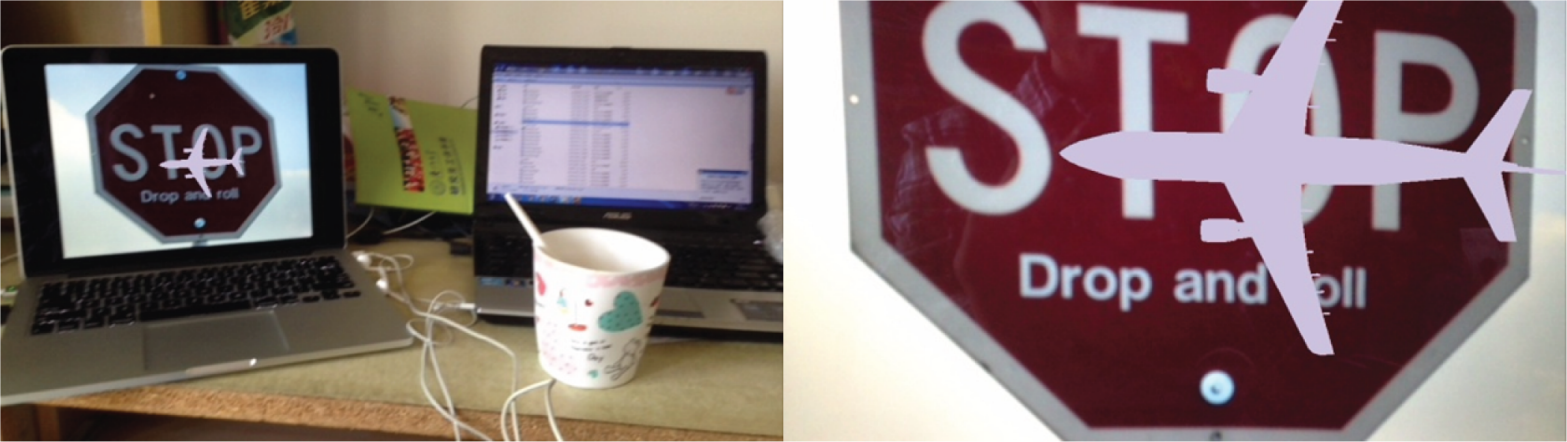
The augmented effect of the system after changing the target scale.

**Fig 25:**
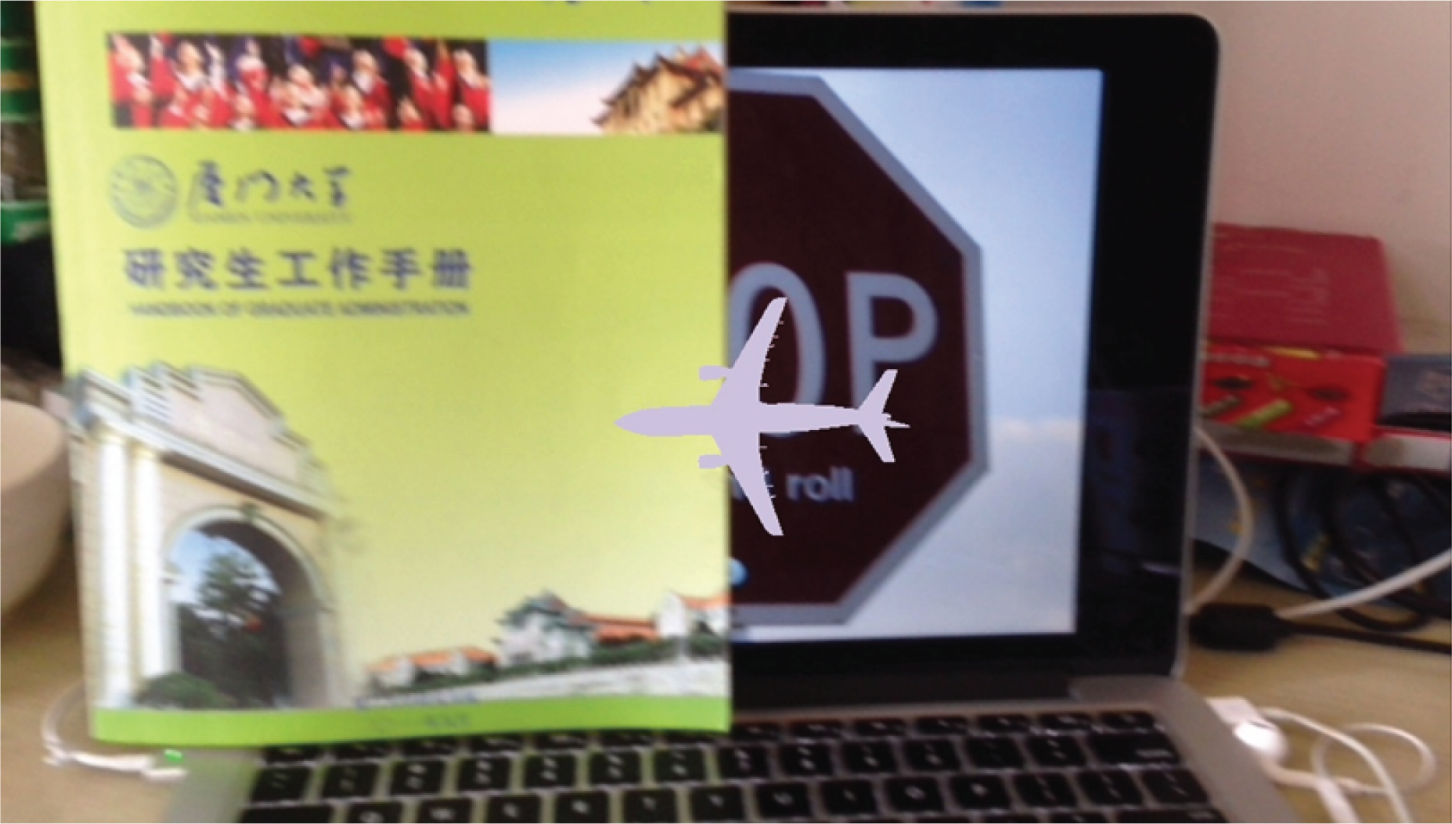
The augmented effect of the system under obstruction condition.

The augmented reality effects of the system under rotation, scale changes and obstruction conditions were presented from Figs 21–25. The results show that the mobile augmented reality system designed in this paper can accomplish the 3D registration of plane model accurately.

Meanwhile, we summarized the time consumption of the system in each module. As shown in Table 5, the performance of the system mainly depends on the feature extraction and matching part. Through the analysis of the data, if each video frame is conducted with feature extraction operation, the augmentation of each video frame usually needs 105-350ms to accomplish. Generally, to assure the fluency of the video when playing, the video playing frequency should be maintained at about 20 frames per second. In other words, the processing time of each video frame is about 50ms. Obviously, only by using the registration method of feature extraction and matching is unable to satisfy the real-time requirement of mobile augmented reality system. Hence, the system in this paper considered to conduct KL tracking registration by taking advantage of video continuity with inertial sensor. Under the premise of affirming target scene, it is unnecessary to conduct the feature positioning and matching for subsequent video frames but use KL algorithm to track current feature position on the basis of the feature and sensor information of the previous frame. For most of the image frames in the video, the registration positioning only needs 10-20ms and processing of each frame needs about 40-70ms, which basically satisfied the real-time requirement of the system.

**Table 5:** Time consumption of mobile augmented reality system module.

Module	Captured Video	Gravity FREAK Feature Extraction and Matching	Inertial KL Track Registration	Plane Model Rendering
**Time Consumed [ms]**	5~10	75~300	10~20	25~40

## Conclusions

With the widespread use of smartphones in recent years, AR application has been transited from PC system to mobile system [39, 40]. Its mobility and real-time problem also brings a lot of challenges. Based on this, this paper proposed the Gravity-FREAK feature extraction algorithm and Gravity-KLT tracking registration algorithm based on MEMS sensor to improve the classic AR algorithm based on PC. Compared with traditional FREAK algorithm, Gravity-FREAK feature descriptor algorithm only needs to use the system interface to read the sensor data when calculating directions, which can save the time of calculating the gray gradient at feature point neighborhood and improve the operating speed of the algorithm. Regarding the defect of traditional KL algorithm’s sensitivity on rapid rotation and a wide range of movement, MEMS sensor can provide initialization parameters for KLT algorithm which would enable the increase of algorithm convergence rate when the camera moves fast and improve the robustness of the registration tracking of mobile augmented reality. In the future, deeper improvements can be made based on this paper. First, improvements can be made on the algorithm effectiveness when the device is positioned at horizontal or approximately horizontal scene. Secondly, on the mechanism of offline registration, dependency on PC offline registration can be further solved by using cloud computing server combined with smartphones to realize remote online registration. Finally, improvements can be made on virtual registration object and visual fusion effect of real scene, including adding more real shadow and illumination effect, restoring the obstruction effect as well as the consistent experience of real world and virtual world to improve the overall user experience of mobile AR system.
